# Drug Repositioning for Effective Prostate Cancer Treatment

**DOI:** 10.3389/fphys.2018.00500

**Published:** 2018-05-15

**Authors:** Beste Turanli, Morten Grøtli, Jan Boren, Jens Nielsen, Mathias Uhlen, Kazim Y. Arga, Adil Mardinoglu

**Affiliations:** ^1^Science for Life Laboratory, KTH Royal Institute of Technology, Stockholm, Sweden; ^2^Department of Bioengineering, Istanbul Medeniyet University, Istanbul, Turkey; ^3^Department of Bioengineering, Marmara University, Istanbul, Turkey; ^4^Department of Chemistry and Molecular Biology, University of Gothenburg, Gothenburg, Sweden; ^5^Department of Molecular and Clinical Medicine, Sahlgrenska University Hospital, University of Gothenburg, Gothenburg, Sweden; ^6^Department of Biology and Biological Engineering, Chalmers University of Technology, Gothenburg, Sweden

**Keywords:** prostate cancer, drug repositioning, non-cancer therapeutics, repurposing, approved drugs

## Abstract

Drug repositioning has gained attention from both academia and pharmaceutical companies as an auxiliary process to conventional drug discovery. Chemotherapeutic agents have notorious adverse effects that drastically reduce the life quality of cancer patients so drug repositioning is a promising strategy to identify non-cancer drugs which have anti-cancer activity as well as tolerable adverse effects for human health. There are various strategies for discovery and validation of repurposed drugs. In this review, 25 repurposed drug candidates are presented as result of different strategies, 15 of which are already under clinical investigation for treatment of prostate cancer (PCa). To date, zoledronic acid is the only repurposed, clinically used, and approved non-cancer drug for PCa. Anti-cancer activities of existing drugs presented in this review cover diverse and also known mechanisms such as inhibition of mTOR and VEGFR2 signaling, inhibition of PI3K/Akt signaling, COX and selective COX-2 inhibition, NF-κB inhibition, Wnt/β-Catenin pathway inhibition, DNMT1 inhibition, and GSK-3β inhibition. In addition to monotherapy option, combination therapy with current anti-cancer drugs may also increase drug efficacy and reduce adverse effects. Thus, drug repositioning may become a key approach for drug discovery in terms of time- and cost-efficiency comparing to conventional drug discovery and development process.

## Introduction

The nature presented itself as a potential drug resource for scientists enabling serendipitous discoveries for centuries. The venture of drug discovery has started with random screening of crude plants such as reserpine, lavender, and opium, and latterly created the drug industry, which is the one of the most advanced industries in our society (Giridhar, 2012). As a part of this industry, the drug discovery process had enormous investments into infrastructures. Research and development (R&D) require huge amounts of time and money. Further, translation of the promising molecule to an approved drug has major problems including high failure rates and withdrawal risks by reason of safety or efficacy problems (Shim and Liu, 2014; Würth et al., 2016).

Drug repositioning (DR), which is an auxiliary process to conventional drug discovery has gained attention from both academia and pharmaceutical companies. Drug repositioning, also named “drug repurposing or drug recycling,” is a promising approach to overcome hurdles in discovering and developing new drugs via identification of the new therapeutic applications of known drugs. Repurposed drug candidates have established formulations and manufacturing methods, extensive pharmacokinetic properties, known adverse effects, clinical trial information, and post-marketing surveillance safety data which make a beeline for the drug development with less chance of failure (Deotarse et al., 2015). To date, there are many success stories about repositioned drugs. The first and most well-known example of DR was discovered serendipitously. Sildenafil was originally produced to be used in coronary artery disease but after the failure on phase 2 trials, it was directed to the treatment of erectile dysfunctions because of its side effect in inducing penile erections (Li and Jones, 2012; Deotarse et al., 2015). Thalidomide, everolimus, nelfinavir, minoxidil, and more drugs have been proven effective in treating another disease than intended at first indications (Huang et al., 2011; Li and Jones, 2012; Pantziarka et al., 2014; Deotarse et al., 2015).

Cancer is one of the most complex diseases as well as the leading cause of death worldwide. Epidemiologic studies pointed out 14.1 million new cancer cases and 8.2 million deaths globally in 2012. Among all cancer types, prostate cancer (PCa) is the fourth most prevalent cancer without distinction of gender and the second most prevalent cancer among men. An approximate 1.1 million cases were diagnosed with PCa in 2012, accounting for 15% of the cancers diagnosed worldwide in men (Ferlay et al., 2015). A recent report showed that PCa in the United States is the first and second leading cancer type considering estimated new cases and deaths, respectively (Siegel et al., 2018).

Most of the conventional anti-cancer chemotherapeutic agents may have notorious adverse effects that drastically reduce the life quality of cancer patients. Therefore, DR is an encouraging strategy to identify non-cancer drugs having anti-cancer activity as well as little or tolerable adverse effects for human health. Hence, DR and any progression on workflows for efficient DR may ameliorate the professed inefficiency of conventional drug development process as previously discussed (Shim and Liu, 2014; Vanhaelen et al., 2017).

In the context of drug discovery, this review focuses on non-cancer drugs that are repurposed to be used as potential PCa therapeutics. We begin by discussing the different DR approaches which enable the researchers to not only discover new candidates but also validate their results as a part of the study. Second, a comprehensive overview is given on the repurposed non-cancer drugs against PCa which have been under clinical investigation until now. Finally, we discuss the gaps and future challenges on drug repositioning approaches and the concepts to propel the field forward for treating PCa as well as other cancer types. Drugs repositioned for PCa treatment may potentially be used in other cancer types since common pathways or targets might be shared in different cancer types regardless of tissue types.

## Drug Repositioning Approaches for the Treatment of PCa

**FIGURE 1:**
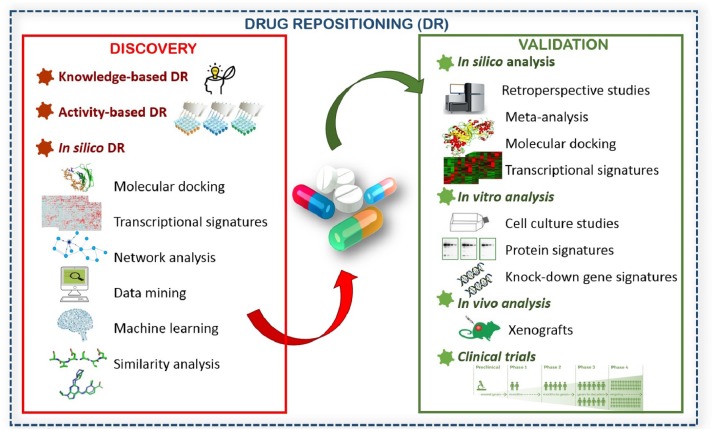
Different drug repositioning approaches for drug discovery and validation.

Drug repositioning approaches are applicable when discovering new therapeutics and also validating the candidate drugs. We evaluated methods used for discovery of new therapeutics in three main categories (**Figure 1**) and explained them in terms of PCa treatment. Activity-based DR and *in silico-*based DR were mentioned previously (Shim and Liu, 2014). However, a notable number of current drug repositioning studies for PCa were established based on observations from previous studies. Therefore, we aimed to consolidate this categorization with knowledge-based DR. We provide examples of DR for PCa comprising different discovery and validation methods (**Table 1**). We also discuss alternative/complementary approaches and their advantages/disadvantages for new drug discovery (**Table 2**).

**Table 1:** Non-cancer drug repositioning candidates for prostate cancer and their repurposing strategies.

Drugs	Original Use	Proposed Anti-cancer Mechanisms	Repurposing Methods	Validation Methods	Reference
Naftopidil	Alfa-blocker	-Akt phosphorylation inhibitory effects	Knowledge-based	*In vitro In vivo*	Iwamoto et al., 2017
		-Inhibiting prostate tumor growth			
Niclosamide	Anti-helminthic agent	-Inducing Wnt co-receptor LRP6 degradation	Knowledge-based	*In vitro*	Lu et al., 2011
		-Inhibiting the Wnt/β-Catenin Pathway			
Ormeloxifene	Estrogen Receptor Modulator	-Inhibition of oncogenic β-catenin signaling and EMT progression	Knowledge-based	*In silico In vitro In vivo*	Hafeez et al., 2017
		-Suppresses prostate tumor growth and metastatic phenotypes			
Nelfinavir	Anti-retroviral agent	-Inhibiting proliferation and inducing apoptosis	Knowledge-based	*In silico In vitro*	Guan et al., 2015
		-Suppression of regulated membrane proteolysis			
Glipizide	Anti-diabetic agent	-Inhibiting angiogenesis	Knowledge-based	*In vivo*	Qi et al., 2016
Ferroquine	Anti-malarial agent	-Inhibiting autophagy	Knowledge-based	*In vitro In vivo*	Kondratskyi et al., 2017
		-Negatively regulation of Akt kinase and HIF-1α			
Nitroxoline	Anti-bacterial agent	-Induced G1 arrest of cell cycle and subsequent apoptosis	Knowledge-based	*In vitro*	Chang et al., 2015
		-Induced autophagy through an AMPK-dependent pathway			
Triclosan	Anti-bacterial agent	-Inhibition of FASN	Activity-based	*In vitro*	Sadowski et al., 2014
		-TCS binds and inactivates the enoyl reductase domain			
Digoxin	Anti-arrhythmic agent	-Causing an influx of intracellular calcium into prostate cancer cells	Activity-based	*In silico*	Platz et al., 2011
		-Triggering apoptosis			
		-Potential HIF-1α inhibitor			
Clofoctol	Anti-bacterial agent	-Activation of unfolded protein response pathways	Activity-based	*In vitro In vivo*	Wang et al., 2014
		-Induction of G1 cell cycle arrest in prostate cancer cells			
Risperidone	Anti-psychotic agent	-Inhibition of 17HSD10 which is an intracellular binding partner for Amyloid b-Peptide and overexpressed in PCa bone metastasis	*In silico*	*In silico In vitro In vivo*	Dilly et al., 2017
Dexamethasone	Anti-inflammatory agent	-Modulator of ERG activity	*In silico*	*In silico In vitro*	Gayvert et al., 2016
Zenarestat	Aldose reductase inhibitor	-Potent inhibitor of NF-κB pathway	*In silico*	–	Turanli et al., 2017


**Table 2:** Different approaches for drug repositioning.

Approaches	Advantages	Disadvantages
Knowledge-based DR	Mainly based on researchers’ know-how	Higher rate of false positive related to the observations
	Time- and cost-efficient process	Limited number of drugs evaluated at once
	Easy to validate in pre-clinical and clinical studies	
Activity-based DR	Lower rate of false positive hits during the experiments	Requires an entire collection of drugs
	Easy to validate in pre-clinical and clinical studies	Time-consuming and costly process
	No limitation for target-based and cell-based screening assays	Requires developing an assay for pre-screening
*In silico* DR	Time- and cost-efficient process	Higher rate of false positive hits during the experiments
	No need an entire collection of existing drugs	Highly dependent on availability of information such as structural information of target proteins, disease phenotype information, etc.
	Easy to integrate different methods	Low accuracy of predicting new drug-target interactions
	Easy to evaluate huge number of drugs at once	


### Knowledge-Based Drug Repositioning

The term “knowledge-based drug repositioning” represents drug repositioning based on knowledge of the medical doctors or researchers and their ability to interpret scientific observations or just coincidences. The first examples of drug repositioning were found serendipitously while working on another disease. Afterward, similar disease indications, sharing altered pathways or the need of combination therapies, led researchers to look for observations and repurpose drugs for a disease rather than the originally targeted one. Hence, these success stories have become prominent and still practicing in the context of drug repositioning. Recent knowledge-based DR studies on PCa have presented new candidates such as ormeloxifene (Hafeez et al., 2017), naftopidil (Iwamoto et al., 2017), ferroquine (FQ) (Kondratskyi et al., 2017), nelfinavir (Guan et al., 2015), nitroxoline (Chang et al., 2015), and glipizide (Qi et al., 2016).

Previous studies showed that loss of E-cadherin accompanying abundancy of N-cadherin is involved in epithelial-to-mesenchymal transition (EMT), leading to PCa being metastatic and more aggressive. Moreover, aberrant expression of β-catenin which plays a major role in EMT is a part of the major oncogenic signaling pathways (Jaggi et al., 2005, 2006). Hafeez et al. (2017) suggested that the clinically approved selective estrogen receptor modulator, ormeloxifene, may inhibit EMT by repressing N-cadherin and β-catenin/TCF-4 transcriptional activity. Ormeloxifene has already been reported to demonstrate anti-cancer activity in different carcinoma such as ovarian, head and neck, and breast. However, Hafeez et al. (2017) studied ormeloxifene for the treatment of PCa and explained its effects on EMT processes and Wnt/β-catenin signaling. To validate their hypothesis, they used molecular docking as an *in silico* validation method and ormeloxifene showed proficient docking with β-catenin and GSK-3β. In addition, *in vitro* cell culture studies showed that ormeloxifene induced apoptosis, and reduced tumorigenic, metastatic, and invasive potential of PCa cells. Moreover, treatment remarkably reduced the prostate tumor growth in the xenograft mouse models as *in vivo* validation for effects of ormeloxifene.

Radiotherapy (RT) is an alternative option to surgery for the treatment of localized PCa. However, cancer cells develop resistance to RT, since RT induces upregulation of anti-oxidant enzymes which decompose free radicals inside the cancer cell and protect them. To increase efficiency of therapy, RT has been investigated in combination with Akt inhibitors (Xu et al., 2010) and, in another study, with a Hedgehog inhibitor (Gonnissen et al., 2016). The selective adrenoreceptor A1D antagonist naftopidil is used for treating lower urinary tract symptoms triggered by benign prostatic hyperplasia (Masumori, 2011). Iwamoto et al. (2017) proposed naftopidil therapy in combination with RT to increase treatment efficiency. They reported significant growth inhibitory effects in addition to Akt phosphorylation-inhibitory effects of naftopidil.

Targeting autophagy was presented as promising direction to repurpose anti-cancer therapeutics and therefore discussed in detail (Piao and Amaravadi, 2016; Rebecca and Amaravadi, 2016), since autophagy is upregulated in many cancers and promotes survival of cancer cells in particular on advanced stages that are under metabolic stress. In addition, lysosomes have a major role in autophagy and effective lysosomal function plays a critical role in tumor invasion, resistance to apoptosis and angiogenesis. In addition to two anti-malarial drugs, chloroquine (CQ) and hydroxychloroquine (HCQ), which have already been under clinical investigation for further use in cancer therapy, Kondratskyi et al. (2017) investigated FQ, another anti-malarial agent. They reported that FQ effectively inhibits autophagy, agitates lysosomal functions, and negatively affects tumor growth *in vivo*. Results showed that even in extreme conditions such as starvation and hypoxia observed in advanced solid cancers, FQ negatively regulates Akt kinase and hypoxia-inducible factor-1α (HIF-1α).

Nitroxoline is a widely used antibiotic for treating urinary tract infections due to its pharmacokinetically long retention time in urine (Zhang et al., 2016). Several target-based and cell-based assays demonstrated nitroxoline as an anti-cancer agent leading to the inhibition of endothelial cell proliferation for different cancer types including lymphoma, leukemia, pancreatic cancer, ovarian cancer (Jiang et al., 2012), bladder cancer (Shim et al., 2010), and breast cancer (Sosiè et al., 2013). Furthermore, nitroxoline could potentially be useful for therapeutic development against PCa, since the drug results in AMPK-dependent inhibition of the mTOR signaling pathway and cyclin D1-Rb-Cdc25A axis, leading to apoptosis (Chang et al., 2015).

Nelfinavir is a FDA-approved human immunodeficiency virus (HIV) protease inhibitor which was used in combination therapy for HIV infected patients. Nelfinavir has also been reported to exhibit distinct anti-cancer mechanisms such as endoplasmic reticulum (ER) stress-unfolded protein response pathway and Akt inhibition in various carcinomas such as ovarian, pancreatic, and breast (Koltai, 2015; Bhattarai et al., 2016) in addition to PCa (Guan et al., 2015). However, even though nelfinavir has been investigated in phase 1/2 clinical trials for solid tumors, Kaposi’s sarcoma and others, it is yet to be evaluated for PCa in clinical trials. The mechanisms of action for anti-tumor properties of nelfinavir appear numerous including inhibition of (PI3K)/Akt signaling pathway, the proteasome and HIF-1α which inhibits angiogenesis, and induction of ER stress, autophagy, and apoptosis (Gantt et al., 2013).

### Activity-Based Drug Repositioning

The need of repurposed drugs is increased to compensate the low success rate of conventional or *de novo* drug discovery processes. On the other hand, the availability of established drug libraries led researchers to create more rational designs compared to knowledge-based drug repositioning. The term “activity-based drug repositioning” stands for testing actual drugs in assays.

To date, besides the ones provided by commercial companies, there are two publicly available comprehensive clinical compound libraries, established through support of government and private agencies (Padhy and Gupta, 2011). The first initiative, Johns Hopkins Clinical Compound Library (JHCCL), was launched in early 2000s as a joint collaboration between Johns Hopkins Pharmacology and Johns Hopkins Bloomberg School of Public Health. JHCCL is the largest publicly accessible collection of existing drugs with approximately 3,100 available compounds, many of which are FDA-approved or approved by its foreign counterparts (Padhy and Gupta, 2011; Shim and Liu, 2014). Second collection was built by NIH Chemical Genomics Center (NCGC) and called NCGC Pharmaceutical Collection (NPC) which possess 2,500 small compounds that have been approved for clinical use by the United States (FDA), European (EMA), Japanese (NHI), and Canadian (HC) authorities (Huang et al., 2011). NPC is also comprehensive, publicly accessible collection of approved compounds along with approximately 1,000 additional investigational compounds for high-throughput screening. There are also commercially available clinical drug collections on the market. These drug collections have been recognized as valuable resources for DR purposes in many academic studies. To get a comprehensive drug library, the compounds (i.e., off-patent drugs) that are easy to obtain in terms of price and patent restrictions are generally collected first. Drugs with patent that covers drug synthesis or use will be the most difficult and expensive to obtain, concerning price per compound. Major drug metabolites should be included into collections to increase the size of the drug library, as most of them often have diverse pharmacological properties. For instance, fexofenadine, which is a non-sedating anti-histamine, lacks the cardiotoxic side effects of its parent terfenadine (Sussman et al., 1999). These comprehensive drug libraries can be used for both protein target-based and cell-based screens which shed a light on clinical trials (Shim and Liu, 2014).

Despite the great potential of the activity-based DR, even the largest publicly available drug collection, JHCCL, covers almost 30% of roughly 10,000 drugs in clinic. Though this seems a large library, it pales in comparison with the libraries of novel compounds (100,000 or more) held by pharmaceutical companies. Availability is another issue for drug screening. JHCCL shared the drug collection with over 45 researchers worldwide for drug screening against different diseases. This existing library is publicly and freely available in principle but requires financing of the replacement of drug stocks and shipping costs (Chong and Sullivan, 2007). In general, drug libraries are provided in 384-well plates and can easily be scaled up. However, unlike the other DR methods, activity-based DR requires specialized robotics to screen thousands of wells containing drugs/compounds.

There are several promising hits identified by activity-based DR including cancers, diabetes, and infectious diseases (Chong et al., 2006a,b, 2007; McMahon et al., 2007; Zhang et al., 2008; Kim et al., 2010; Shim et al., 2010; Lin et al., 2011; Platz et al., 2011). Among the hits, anti-fungal agent, itraconazole, and cardiac glycoside, digoxin are promising anti-cancer agents that have been explored through activity-based DR using JHCCL (Chong et al., 2007; Zhang et al., 2008). To date, phase 2 clinical studies evaluating the anti-tumor efficacy of itraconazole were completed for treatment of metastatic non-squamous non-small cell lung cancer (Rudin et al., 2013) and metastatic PCa (Antonarakis et al., 2013). Digoxin was found to have significant effect both at the epidemiological and experimental levels (Platz et al., 2011) and clinically trialed in PCa cases (Lin et al., 2014).

Triclosan [5-chloro-2-(2,4-dichlorophenoxy) phenol] is effective against many different bacteria as well as some fungi and protozoa. It has been widely used as a broad-spectrum anti-bacterial and anti-fungal agent for more than 30 years. It is found in formulations as diverse as cosmetics, fabrics, toothpastes, plastic kitchenware, and toys. In previous studies, triclosan demonstrated blocking effect on lipid synthesis (McMurry et al., 1998; Levy et al., 1999). Deregulated lipid metabolism and FASN inhibition has emerged as a promising therapeutic target in cancer patients. Sadowski et al. (2014) inhibited FASN and compared the cellular and molecular effects of triclosan in both malignant and non-malignant prostate cells compared to the other well-known FASN inhibitors C75 and orlistat. As a result, they proposed triclosan as a promising drug candidate against PCa with a superior cytotoxic profile in comparison with C75 or orlistat.

Clofoctol is an antibiotic used in the therapy of upper respiratory tract infections. It was previously reported to inhibit protein translation in mammalian cells and it is a potential anti-cancer drug (Pelletier et al., 2007). Wang et al. (2014) demonstrated a quite unique mechanism of action of clofoctol that inhibits prostate tumor growth as well as protein translation in PCa cells. It has indirect effect via inducing ER stress and activating unfolded protein response pathways which serve a cellular protective mechanism to cope with an excess of misfolded proteins in the ER.

### *In Silico* Drug Repositioning

*In silico* drug repositioning is a potentially powerful strategy since data acquired from structural biology and omics technologies have been accumulating in the past few decades. *In silico* DR provides a faster repurposing process as well as reduced costs through different approaches such as molecular docking, network analysis, data mining, similarity analysis, machine learning, and transcriptional signatures (**Figure 1**). Different methods covered by *in silico* DR has been recently reviewed in detail (Vanhaelen et al., 2017).

In recent years, *in silico* DR methods have been focused on identification of candidate targets and potential drugs considering data from different omics levels as well as the disease-gene-drug triad (Sun et al., 2017). Publicly available clinical data at genomic and/or transcriptomic levels were evaluated within a comprehensive, systematic, and integrative analysis pipelines in several studies to find out new drug candidates for particular medical problem (Sirota et al., 2011; Jin et al., 2012; Turanli et al., 2017). Computational predictions supported by experimental assessments have been effectively used to determine new DR potentials. In contrast, powerful computational resources such as software for retrospective analysis or structural biology and maintenance of web-based databases are decisive for the collection and evaluation of the experimental data (Vanhaelen et al., 2017).

Risperidone, a benzisoxazole derivative, is an anti-psychotic agent which has proven efficacy in the treatment of most common central nervous system disorders such as depression, bipolar disorder, and schizophrenia (Roth et al., 2004). Although the drug mechanism is not clear, it has been reported that it has a high binding affinity for both dopamine D2 and serotonin 5-HT2 receptors (Abou El-Magd et al., 2010). Another molecular target of risperidone, 17-b-hydroxysteroid dehydrogenase 10 (17HSD10), plays a significant role as an intracellular binding partner for amyloid β-peptide which is important in Alzheimer’s disease. Additionally, it is suggested for treatment of PCa to stimulate dihydrotestosterone synthesis in the absence of testosterone and also highly expressed in PCa bone metastases (Ayan et al., 2012). Recently, Dilly et al. (2017) reported risperidone as a DR candidate targeting 17HSD10. They combined both activity-based and *in silico* DR methods through the discovery process, and then tested other similar drugs in addition to risperidone *in silico*, *in vitro* and *in vivo* through validation process.

Acetic acid derivative, zenarestat is an aldose reductase inhibitor originally used for preventing diabetic cataractogenesis, retinopathy, and neuropathy (Schemmel et al., 2010; Zhu, 2013). Since aldose reductases activate the transcription of NF-κB and AP-1 which transcribe the genes of inflammatory cytokines and oxidative stress-induced inflammation which promotes the formation of cancerous tumors, recent investigations strongly suggest that the use of aldose reductase inhibitors can be novel chemotherapeutic agents against cancer (Tammali et al., 2011). As an *in silico* DR application, the web-based DR tool, geneXpharma, has taken into consideration comprehensive gene expression profiling and the disease-gene-drug association data and repurposed zenarestat as a candidate drug to be used in PCa treatment at the point of experimental validation (Turanli et al., 2017).

As always, every computational approach has its own advantages and limits based on the problem to be solved and on the type, quality, and quantity of information available about the problem in the literature or in public/commercial databases. As an example, molecular docking requires high resolution structural information of drugs and targets (Shim and Liu, 2014). The integration of different computational methods and information coming from different sources such as biological, structural, and clinical databases could serve valuable opportunities in the manner of purposing new use of an old drug (March-Vila et al., 2017). However, none of these methods are sufficient enough to model the complex interactions among drugs, targets and diseases. Keeping this in mind, *in silico* drug repositioning could represent a primary method in order to highlight novel small molecules or drug compounds through initiating with a hypothesis or generating new hypotheses about how these compounds work and what their primary targets are and eventually, such hypotheses may drive the systematic design of downstream experiments. Although there are plenty of computational methods, their integration can surpass the methods applied alone.

After brief explanations of each perspective, it sounds that any of them has their own pros and cons as summarized in **Table 2**. Knowledge-based DR is solely dependent on researches’ knowledge and ability to interpret the scientific observations or just coincidences. It is not only a time- and cost-efficient process, but also easy to validate in pre-clinical and clinical studies if observations were experimentally investigated. On the other hand, it is not the systematic way comparing to the other DR methods, and therefore it may appear risky. *In silico* systems has reduced time and cost, however, it always hinges on the availability of the experimental data such as structure or gene expression profiles at first. The biological significance of the putative targets predicted by the computationally method must also be experimentally assessed. On the other hand, activity-based DR is time- and labor-consuming; requiring an entire collection of existing drugs, specialized equipment and develop a screening assay, but it can be employed without requiring structural information of target proteins or database. Moreover, activity-based DR is easy to validate since it experiences low false positive rates with regard to *in silico* repositioning (Shim and Liu, 2014).

## Repurposed and Clinically Evaluated Non-Cancer Drugs for PCa Treatment

**FIGURE 2:**
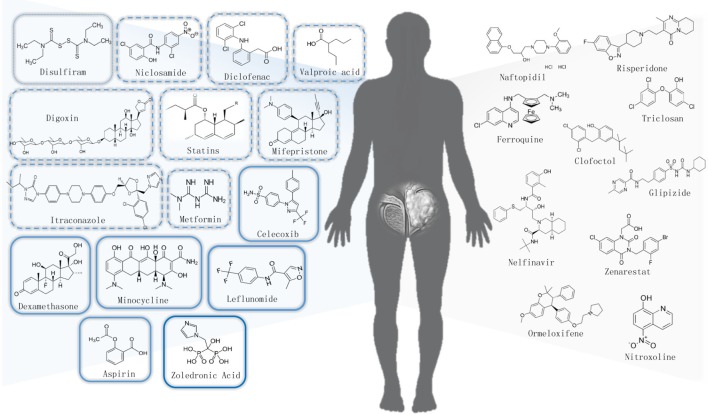
Chemical structures of repurposed non-cancer drug candidates for prostate cancer.

We briefly summarize the non-cancer approved drugs which are repurposing for PCa treatment via different DR approaches in **Table 1**. Moreover, chemical structures of all potential non-cancer drugs against PCa were shown in **Figure 2**. Here, we provide an overview only on existing drugs with anti-PCa activities that are under clinical investigation as reviewed in **Table 3**.

**Table 3:** Non-cancer drug repositioning candidates under clinical investigation for the treatment of prostate cancer.

Drugs	Original Use	Proposed Anti-cancer Mechanisms	Phase	Identifier^∗^	Recruitment Status
Zoledronic Acid	Bisphosphonate	Inhibition of mevalonate pathway Activity of metalloproteinases	Phase 4	NCT00219271	Completed
Dexamethasone	Anti-inflammatory agent	Modulator of ERG activity	Phase 3	NCT00316927	Completed
Aspirin	Anti-inflammatory agent	COX inhibitor suppression of the neoplastic prostaglandins Inhibition of NF-κB	Phase 3	NCT00316927	Completed
Minocycline	Anti-bacterial agent	Inhibition of pro-inflammatory cytokines Inhibition of matrix metalloproteinases	Phase 3	NCT02928692	Recruiting
Celecoxib	Anti-inflammatory agent	Selective Cox-2 inhibitor Inhibition of NF-κB activity Inhibition of PDPK1/Akt signaling pathway	Phase 2/3	NCT00136487	Completed
Leflunomide	Immunomodulatory agent	Potent inhibitor of tyrosine kinases	Phase 2/3	NCT00004071	Completed
Statins	HMG-CoA reductase inhibitors	Inhibition of mevalonate pathway	Phase 2	NCT01992042	Completed
Metformin	Anti-diabetic agent	PI3K/Akt/mTOR signaling pathway Triggering apoptosis Inhibition of hepatic gluconeogenesis	Phase 2	NCT03137186	Recruiting
Valproic acid	Anti-epileptic agent	Histone deacetylases inhibitor Inhibition of SMAD4 expression	Phase 2	NCT00670046	Unknown
Diclofenac	Anti-inflammatory agent	Inhibition of COX-2 and prostaglandin E2 Inhibition of MYC	Phase 2	NCT01939743	Completed
Niclosamide	Anti-helminthic agent	Targeting multiple signaling pathways (NF-κB, Wnt/β-catenin, Notch, ROS, mTORC1, and Stat3)	Phase 2	NCT02807805	Recruiting
Itraconazole	Anti-fungal agent	Anti-angiogenic Hedgehog pathway inhibition Autophagy induction	Phase 2	NCT00887458	Completed
Digoxin	Anti-arrhythmic agent	Inhibit HIF-1α synthesis Inhibition of TNF-α/NF-κB pathway Activation of Src kinase pathway	Phase 2	NCT01162135	Completed
Mifepristone	Anti-progestational agent	blocking cell surface receptors on solid tumors	Phase 2	NCT00140478	Completed
Disulfiram	Alcohol deterrent agent	DNMT1 inhibitor Induction of metallothionein expression	Phase 1	NCT02963051	Recruiting


*^∗^Identifiers were sourced by https://clinicaltrials.gov.*

### Dexamethasone

Dexamethasone is one of the glucocorticoids that prevent inflammation and has immunosuppressive activity. It serves potential to treat several different diseases such as allergic disorders, inflammatory disorders (ulcerative colitis, arthritis, lupus, psoriasis), or breathing disorders (Buttgereit et al., 2011). Dexamethasone in combination with other chemotherapeutic drugs is used to counteract certain side effects of chemotherapy such as edema, emesis, etc. For instance, it is used to stabilize the development of edema in brain tumors, which could eventually compress other brain structures or spinal cords (Chu et al., 2014). The antiemetic action of dexamethasone reduces pain and the concomitant use of opioids, which in turn reduces opioid-related nausea and vomiting (Chu et al., 2014). Besides, dexamethasone can be used alone as a direct chemotherapeutic agent in certain hematological malignancies, especially in the treatment of multiple myeloma (Bhattarai et al., 2016). Although combination of dexamethasone with chemotherapeutic agents were already clinically tested in PCa patients (Shamash et al., 2011), oncogenic transcription factors were also targeted via systematic drug-transcription factor. Analysis predicts the candidate small molecules in one recent *in silico* DR study, and they showed the effect of dexamethasone on prostate tumor by inhibiting ERG Activity (Gayvert et al., 2016).

### Aspirin

Acetylsalicylic acid or aspirin is used as an analgesic and non-steroidal anti-inflammatory drug (NSAID) in a variety of conditions ranging from headache, fever associated with the common cold, pain in rheumatoid arthritis and other similar musculoskeletal conditions (Kar, 2007).

It is known as a cyclo-oxygenase enzyme (COX-1 and 2) inhibitor with a great affinity (Yoshimura et al., 2000). To date, the NSAIDs such as aspirin, which inhibits both COX-dependent and/or independent pathways has been speculated to be used against some malignancies including colorectal, esophageal cancer, breast as well as prostate (Downer et al., 2017). Particularly, COX-2 which plays role in carcinogenesis, angiogenesis, poorer prognosis in PCa (Liu et al., 2014), and inhibition of immune surveillance (Kirschenbaum et al., 2001) is highly expressed in malignant prostate tissue compared to benign human prostate tissue. Anti-cancer mechanism of aspirin is the inhibition of COX-2 which leads to suppression of the neoplastic prostaglandins production. In addition to its classical role as COX inhibitor, it suppresses activation of NF-κB induced by TNF, upregulation of tumor suppressor gene, and downregulation of anti-apoptotic gene (Bhattarai et al., 2016).

To date, several epidemiologic studies and meta-analyses of prospective and case-control cohort studies have discussed the use of NSAID as well as aspirin in PCa cases worldwide and reviewed in detail (Fowke et al., 2009; Mahmud et al., 2010; Liu et al., 2014; Vidal et al., 2015; Bilani et al., 2017). However, results were inconsistent since some studies suggested a reduced risk of total PCa and PCa-specific mortality in association with NSAID use, some reported no benefit or elevated risk of PCa (Assayag et al., 2015). Liu et al. (2014) indicated the effect of any NSAID on PCa varied by geographic region. North American studies reported a decreased risk of PCa (Vidal et al., 2015), whereas studies from Europe found elevated risk due to the use of any NSAID. Potent effect of aspirin for PCa detection were also addressed in the context of PCa prevention (Fowke et al., 2009).

### Diclofenac

Diclofenac is another anti-inflammatory and analgesic from the NSAID family and it functions as an inhibitor of COX-2 and prostaglandin E2 synthesis like aspirin. Combination or monotherapies of diclofenac have been reported to have positive effects on treating multi cancer types including ovarian cancer, colorectal cancer, neuroblastoma, and melanoma (Pantziarka et al., 2016).

High expression of MYC is characterized in many human malignancies and it leads to the upregulation of glycolytic enzymes such as glucose transporter-1 (GLUT1) and lactate dehydrogenase-A (LDHA) resulting high rates of glucose uptake and glycolysis. Therefore, MYC is a promising target for cancer therapy. COX-independent effect of diclofenac has been proposed that it represses MYC and regulates glucose metabolism via significantly decreased GLUT1 and LDHA gene expression in melanoma, PCa, leukemia cell line proliferation *in vitro*, and reduced melanoma growth *in vivo* (Gottfried et al., 2013). As a conventional COX inhibitor, diclofenac augmented the effect of RT on PCa and proposed as a potential radiosensitizer for treatment of PCa according to *in vitro* and *in vivo* outcomes (Inoue et al., 2013).

### Celecoxib

Celecoxib is another anti-inflammatory drug used for the treatment of adult arthritis and unlike aspirin and diclofenac, it is a selective COX-2 inhibitor (Davies et al., 2000). Since COX-2 is expressed in the malignant epithelial cells as well as the tumor-feeding neovasculature, celecoxib is a promising candidate for treating cancer. Additionally, long-term use of NSAIDs inhibiting both COX-1 and COX-2 have been known to inhibit the production of COX-1-derived prostaglandins required for preservation of gut, kidney, and platelet functions (Leahy et al., 2002). Celecoxib have been reported not only in the treatment of human epithelial tumor types, such as colorectal, breast, and non-small cell lung cancers as well as prostate (Elder et al., 2000; Harris et al., 2000; Hsu et al., 2000), but also in the prevention of colorectal cancer in high-risk patients with familial adenomatous polyposis linked to the APC oncogene mutation (Ponder, 1997).

The main anti-cancer mechanism of celecoxib is to decrease prostaglandin production in the COX-2 expressed tumors, resulting in an inhibition of the proliferation and induction of apoptosis. Moreover, anti-cancer mechanism of celecoxib via COX-2-independent pathway has been reported such as inhibition of PDPK1/Akt signaling pathway, inhibition of NF-κB activity, alteration of APC/β-catenin pathway, and mitochondrial apoptosis pathway (Kulp et al., 2004; Patel et al., 2005; Grösch et al., 2006).

The effect of celecoxib on the expression of anti-apoptotic protein Bcl-2 as well as anti-apoptotic kinase Akt were examined. Results demonstrated inhibition of Akt activation and induced apoptosis independent from Bcl-2 via LNCaP and PC-3 cells *in vitro* (Hsu et al., 2000). Celecoxib and its analog that does not inhibit COX-2 were investigated in PC-3 and DU-145 *in vitro* and PC-3 tumor xenograft *in vivo* resulting inhibition of PDPK1/Akt signaling pathway (Kulp et al., 2004). Findings from *in vivo* and *in vitro* studies suggested that celecoxib has significant COX-2-independent anti-tumor properties which makes it active even in tumors that do not express significant amounts of COX-2. PCa cell growth was inhibited by inducing a G1 cell cycle block and reducing DNA synthesis (Patel et al., 2005). Recently, celecoxib and zoledronic acid were considered complementary targeting clinically later-stage disease (Mason et al., 2017).

### Minocycline

Minocycline is a semi-synthetic tetracycline derivative that maintains the efficacy against both Gram-positive and Gram-negative bacteria. It has been approved by FDA, more than 30 years ago, for the treatment of acne and some sexually transmitted diseases. Subsequently, experimental models revealed non-antibiotic properties of minocycline and determined minocycline beneficial against various disorders with an inflammatory basis such as dermatitis, periodontitis, atherosclerosis, and autoimmune diseases such as rheumatoid arthritis, inflammatory bowel disease (Garrido-Mesa et al., 2013).

Minocycline inhibits the action of proinflammatory cytokines and matrix metalloproteinases (MMPs) which are extracellular matrix-degrading enzymes implicated in cancer invasion and metastasis. Up until now, the drug presents anti-cancer activities against various cancer cell lines, such as ovarian cancer, glioma, PCa, melanoma, and breast cancers (Lokeshwar, 2011; Ataie-Kachoie et al., 2013; Garrido-Mesa et al., 2013). Moreover, minocycline has been in phase 2 of clinical trials for different carcinomas such as myeloma, esophageal, pancreatic, colorectal, lung, head, and neck cancers (Bhattarai et al., 2016) whereas in phase 3 of clinical trials for PCa.

Lokeshwar (2011) investigated doxycycline, minocycline, and chemically modified tetracycline (CMT) analogs for treatment of PCa. Some of these CMTs, notably, CMT-3 and CMT-308, were found significantly more potent than doxycycline or minocycline in inhibiting tumor cell-derived MMPs and inducing apoptosis *in vitro* and *in vivo*. In another study, pharmacokinetic interactions for minocycline with retinoic acid metabolism were investigated in various PCa cell lines (Regen et al., 2014).

### Niclosamide

Niclosamide, the salicyclamide derivative, is an oral anti-helminthic drug to treat tapeworm infections. In addition to be used as an anti-infective agent afterward, it has been demonstrated to exhibit anti-cancer activity in several cancers such as colorectal cancer (Sack et al., 2011), ovarian cancer (Yo et al., 2012), acute myeloid leukemia (AML) (Jin et al., 2012), and breast cancer (Wang et al., 2013). Niclosamide targets multiple signaling pathways which are closely involved with oncogenesis and oncoprogression such as NF-κB, Wnt/β-catenin, Notch, and mTORC1 (Pan et al., 2012).

Niclosamide targets the Wnt co-receptor LRP6 on the cell surface and that it is potent molecule as a Wnt/β-catenin signaling and anti-cancer agent for human prostate and breast cancer due to *in vitro* studies (Lu et al., 2011). Androgen receptor (AR) variants that drives resistance to chemotherapeutics such as enzalutamide are another problem that faced through PCa treatment. Therefore, niclosamide was evaluated as potential inhibitor of variant alternatively spliced AR (AR-V7) and preclinical validation proved that it is promising to treat, either alone or in combination with current anti-androgen therapies in advanced PCa patients (Liu et al., 2014).

### Itraconazole

Itraconazole was a common triazole anti-fungal agent and used to inhibit the cytochrome P450-dependent lanosterol 14α demethylase (14DM). The drug causes the destruction of the fungal membrane and reduction of ergosterol synthesis. However, the anti-fungal effect of itraconazole is unlikely to be associated with its potential anti-cancer activities. It can reverse chemoresistance mediated by P-glycoprotein, adjust the signal transduction pathways of Hedgehog and Wnt/β-catenin and inhibit angiogenesis (Kim et al., 2010; Tsubamoto et al., 2017). The benefits of itraconazole monotherapy for PCa and basal cell carcinoma were proposed in clinical trials as well as *in vitro* studies. Besides, combination of itraconazole and chemotherapy showed the better survival rates in various cancer types such as pancreatic, ovarian, and triple negative breast via reducing endothelial cell proliferation and migration (Chong et al., 2007; Aftab et al., 2011; Tsubamoto et al., 2017). Several possible multi-drug combinations have been outlined and are waiting for clinical trials whereas some of these are on-going. Itracanozole is addressed as promising drug candidate for rapid implementation of clinical trials in rare cancers including medulloblastoma, or certain types of sarcomas (rhabdomyosarcoma, chondrosarcoma, and osteosarcoma) in which the Hedgehog pathway has an important role (Pantziarka et al., 2015).

Xu et al. (2010) suggested that itraconazole inhibits the mTOR and VEGFR2 signaling pathways in endothelial cells by damaging cholesterol trafficking. Afterward, phase 2 study evaluating the anti-tumor efficacy of oral itraconazole in men were completed for treatment of metastatic CRPC. They suggested that high-dose itraconazole (600 mg/day) might have modest anti-cancer activity associated with Hedgehog pathway suppression (Antonarakis et al., 2013).

### Digoxin

Digoxin, as a cardiac glycoside, has basically steroid-like structure including an unsaturated lactone ring and sugar moieties. It affects cardiac contractility through highly specific inhibition of the Na^+^/K^+^-ATPase pump. Anti-cancer activity of digoxin may include intracellular decreased level of K^+^ and increased level of Na^+^ and Ca^2+^ and also inhibits DNA topoisomerase II, IL-8 production, HIF-1α synthesis, and the TNF-α/NF-κB pathway and activate the Src kinase pathway (Slingerland et al., 2013; Calderón-Montaño et al., 2014). Digoxin and its derivatives have been investigated via preclinical studies in several cancer types comprising lung, pancreatic, melanoma, leukemia, renal carcinoma, and the most revised indications were on breast and PCa (Bhattarai et al., 2016).

After an activity-based DR study in which JHCCL was used, 20 drugs were highlighted whereas 2 of them were known HIF-1 inhibitors (rapamycin and roteone) and 11 of these 20 drugs were cardiac glycosides including digoxin, digitoxin, ouabain, etc. As a finding of this study, digoxin treatment increased latency and decreased tumor growth in mice. Digoxin was first reported as an inhibitor of HIF-1α protein and HIF-1 targets such as VEGF, GLUT1, HK1, and HK2 for PCa (Zhang et al., 2008). On the contrary, Lopez-Lazaro (2009) argued that neither digoxin has any inhibitory effect on HIF-1 at therapeutic concentrations, nor the anti-cancer effects observed in mice are relevant in humans. As a result of *in vitro* drug screening using the same drug library, digoxin has emerged again with inhibitory effect on PCa. As a following study, evaluation of a large-scale cohort study with male patients who had heart diseases and took digoxin over 5 years were indicated significantly reduced incidence among PCa patients who have used it compared with the control group (Platz et al., 2011).

### Valproic Acid

The short chain fatty acid, valproic acid (VPA), is an inhibitor of class I histone deacetylases (HDAC) used to treat epilepsy, bipolar disorders, migraine, and schizophrenia. Approximately 20 years ago, the first clinical trial discussing VPA was initiated and then, several clinical trials for different leukemia, and solid tumor entities were followed. In addition to clinical assessments, VPA is still under experimental investigation regarding the numerous mechanisms of anti-cancer activity such as inhibition of tumor-associated inflammation, cancer cell proliferation, tumor angiogenesis, and cancer cell invasion and migration resulting with metastasis and lastly, inhibition of histone deacetylases which plays a central role. Since DNA-region of key anti-cancer genes are tightly bound via histone deacetylase, VPA, as a HDAC inhibitor, allocates silenced genes to be initiated (Michaelis et al., 2007).

Acute and chronic administration of VPA were investigated for PCa cells irrelevant to their androgen sensitivity and also *in vivo* tumor xenograft models. Acute treatment increased net histone H3 acetylation and resulted with upregulated expression of p21, AR, and cytosolic prostate-specific antigen (PSA) where the effects on AR and PSA were reversed for chronically administered VPA in which the cancer cell proliferation rate was decreased due to increased caspase-2/caspase-3 activation and also demonstrated statistically significant reduction of tumor xenograft growth *in vivo* (Xia et al., 2006). In phase 2 trial to treat patients with castration-resistant PCa (CRPC), oral VPA was used with biomarker assessment strategy to study relationship among PSA, serum testosterone, VPA blood levels, and histone acetylation. They exerted toxicities regarding oral use of VPA in the treatment of CRPC patients (Sharma et al., 2008). Lee and Kim (2015) exerted that VPA is more effective on cell viability and invasion in metastatic prostate cell line (PC-3) by upregulating the metastasis suppressor protein NDRG1. VPA is used for the monotherapy of some cancers such as metastatic neuroendocrine carcinomas and myeloid malignancies, however, the most of potent anti-cancer activity of VPA is based on combination with other epigenetic modifiers (azacytidine, hydralazine), combinations with cytotoxic chemotherapy agents (5-FU, epirubicin, cyclophosphamide, doxorubicin, karenitecin), and combinations with immune-modulators (Brodie and Brandes, 2014). In another study, VPA inhibited the expression of SMAD4 which plays an important role in cancer metastasis via EMT along with upregulated the expression of miR-34a in prostate carcinoma (Xia et al., 2016). Very recently, the combination of metformin and VPA were reported to induce synergistic apoptosis in the presence of p53 and androgen signaling; inhibited proliferation of LNCaP and PC-3 cell lines and killed more PCa cells than either drug alone (Tran et al., 2017).

### Statins

Statins are lipid-lowering agents that act by inhibiting 3-hydroxy-3-methylglutaryl (HMG-CoA) reductase resulting with inhibition of the mevalonate pathway. Statins reduce cholesterol levels and are mainly used for the treatment of hypercholesterolemia as well as atherosclerotic plaques causing cardiovascular problems, such as myocardial infarction, stroke, and atrial fibrillation (Hindler, 2006). The emerging interests in the use of statins as anti-neoplastic agents are based on both *in vitro* and *in vivo* studies demonstrating anti-proliferative, anti-metastatic, RT-sensitizing, and apoptosis inducing properties (Chan et al., 2003). Preclinical evidences show that statins inhibit tumor growth and induce apoptosis in melanoma, glioma, neuroblastoma, and leukemia cell lines whereas there are numerous clinical trials that have considered anti-cancer activity of statins in various cancer types such as liver, gastric, acute myeloid leukemia, and squamous cell carcinoma of the cervix and of the head and neck (Chan et al., 2003; Hindler, 2006).

Statin use for the treatment of PCa has been associated with decreased risk of PCa, better prognosis, reduced volume of PCa and PSA levels, along with its protective effect against PCa regarding a retrospective cohort study of men who underwent prostate biopsy (Tan et al., 2011). Numerous studies were in harmony about the lowering PSA effect of statins. Since increased levels of PSA are a common symptom guiding biopsy, reduced PSA levels of statin users were debated in manner of PCa detection in men. Tan et al. (2011) reported no differences on PSA levels of PCa diagnosed patients regardless of statin use and suggested that statin reduces PSA in benign tissue but not in malignant tissues. Additionally, some studies have demonstrated a beneficial effect of statins in reducing PCa related mortality as well as overall PCa risk (Katz et al., 2010; Marcella et al., 2012) whereas other studies have not revealed a significant effect (Caon et al., 2014). Recently, conflicting data sourced from clinical outcomes obstruct the integration of neither beneficial nor harmful associations between the statin use and PCa (Hutchinson and Marignol, 2017).

### Mifepristone

Mifepristone (RU-486), a steroid synthesized in the laboratory, is the first anti-progesterone drug with high affinity for the progesterone receptor (PR) and used for termination of early pregnancies. Since progesterone is crucial for the initiation and maintenance of pregnancy, PR also plays an important role in the human reproductive system (Hazra and Pore, 2001).

Due to knowledge of the overabundance of PR in solid tumors, anti-progesterone drugs have been purposed for tumor growth suppression via blocking cell surface receptors, such as PR as well as glucocorticoid receptors (GR) and ERs (Yu et al., 2015). Cancer cell lines from several tumors such as glioma, breast, prostate, ovary, cervix, and osteoblastoma have been treated with mifepristone and the inhibition of the cancer cell growth regardless of reproductive or non-reproductive origin have been reported (Tieszen et al., 2011).

To the best of our knowledge, one of the earliest studies on anti-progesterone administration to PCa reported *in vivo* suppression of the growth of the androgen-insensitive prostatic carcinoma within combined estrogen therapy (Mobbs and Johnson, 1991). Later, considerable anti-tumor activity of mifepristone was presented in both androgen-sensitive and androgen-insensitive variants of the LNCaP via mouse models (El Etreby et al., 2000). In addition, mifepristone was also assessed in patients with CRPC through phase 2 study. The combination therapy with promising agents such as corticosteroids, ketoconazole, or 5-reductase inhibitors might be used to block the compensatory rise in adrenal androgens and be effective in patients with CRPC (Taplin et al., 2008).

### Disulfiram

Disulfiram is the first FDA approved alcohol aversive drug used for the treatment of alcohol dependence for more than 60 years. It inhibits the aldehyde dehydrogenase (ALDH) leading to accumulation of acetaldehyde in blood (Kalra et al., 2014). As a result of high-throughput cell-based drug screening, disulfiram was found as an anti-cancer drug regarding to reduce tumor growth *in vivo* and induced metallothionein expression. Additionally, the effect of the drug was enhanced by copper *in vitro* (Iljin et al., 2009). To date, numerous clinical trial studies on administration of disulfiram were carried out to treat various cancers such as breast, pancreas, prostate, brain, lung, melanoma as well as metastatic types of some of them.

Disulfiram as acting like a DNA methyltransferase (DNMT1) inhibitor provide advantage by restoring tumor suppressor genes and leading DNA demethylation in PCa cells. It has been linked to reductions in 5-methyl cytosine (5meC) content and decreased methylation in APC and RARB gene promoters (Lin et al., 2011). As follows, disulfiram has been assessed as a potential epigenetic therapy, a pilot trial (NCT01118741) was carried out to evaluate the effect of disulfiram on demethylating changes with biochemically recurrent PCa via quantifying changes in 5meC content in peripheral blood mononuclear cells (PBMC) (Schweizer et al., 2013).

Disulfiram treatment alone showed only minimal effects in castration-resistant PCa xenografts, however, drug supplemented with Cu^2+^ significantly reduced tumor growth (Safi et al., 2014). Very recently, Skrott et al. (2017) reported that the ditiocarb (disulfiram metabolite) and copper complex has anti-cancer effects *in vitro* and *in vivo*. Moreover, NPL4 protein has been proposed a molecular target which is involved in protein turnover of tumorigenesis promoting stress-response pathways.

### Leflunomide

Leflunomide is a selective inhibitor of the rate limiting step in the *de novo* synthesis of pyrimidines. Teriflunomide (A77 1726) is the active metabolite of leflunomide which suppress dihydroorotate dehydrogenase (DHODH) activity as well as IL-1 and TNF-α. It is used mainly for rheumatoid arthritis due to its anti-inflammatory effects (Breedveld and Dayer, 2000).

Additionally, leflunomide has been suggested as a potent inhibitor of tyrosine kinases and some growth factor receptors such as EGFR, FGFR, and PDGFR. Since their activation is associated with numerous cancer types, leflunomide has the potential to be used as an anti-cancer agent (Gupta et al., 2013). One of the earliest studies in which suggested anti-tumor activity of leflunomide showed the inhibition of cancer cells including glioma, ovarian, and prostate which express PDGFR in contrast to cancer cell cultures which only express EGFR (Shawver et al., 1997). Similarly, the active metabolite of leflunomide, teriflunomide, was found more effective at inhibiting the tyrosine kinase activity of PDGFR than that of EGFR and had no effect on the FGFR (Xu et al., 1999).

In a phase 2 trial with leflunomide against hormone resistant PCa, PDGFR expression was noticed in more than 80% of both metastases and primary PCas. Monotherapy of leflunomide demonstrated PSA reduction of more than 50% of patients and also stated significant improvement of pain (Ko et al., 2001). In another study, preliminary results represented chemopreventative effects of leflunomide in PCa (Hail et al., 2010). Mono- and combination therapy of leflunomide is in phase 2/3 of clinical stage for PCa and brain and central nervous system.

### Metformin

Metformin is an anti-diabetic agent primarily being used in type 2 diabetes and increasingly in polycystic ovary syndrome treatment. The direct effect of metformin at the molecular level is to activate AMPK through a tumor suppressor, LKB-1, resulting in decreased glucose level in blood. Moreover, AMPK inhibits mTOR which is key mediator of frequently deregulated PI3K/Akt signaling pathway in malignant cells and activates tumor suppressor p53 leading to cancer cell apoptosis. Indirect effect of the drug is inhibition of hepatic gluconeogenesis and reduces insulin resistance in peripheral tissue. Since tumor cells express high levels of the insulin receptor, reduced insulin levels associated with metformin use provide better prognosis for a number of cancers, including breast, colon, and PCa (Evans et al., 2005; Whitburn et al., 2017). Metformin were reported as an anti-cancer agent against breast, prostate, lung, cervix, and ovarian cancer cells, up to the date. It is also in phase 1–4 clinical trials for various cancers such as lung, prostate, breast, liver, pancreatic, and brain (Jalving et al., 2010).

First epidemiological studies demonstrated metformin as a potent anti-cancer agent in reducing cancer incidence and mortality considering numerous anti-neoplastic biological effects via a range of molecular mechanisms. Some studies supported the oncogenic benefit of metformin for PCa patients regardless their diabetic status (Noto et al., 2012), some of them emphasized heterogeneous and biased results or no significant effects on incidence of PCa (Margel et al., 2013). Since there are plenty of investigations, the current evidences were recently summarized a role for metformin in PCa therapy (Whitburn et al., 2017). Besides, Electronic Health Records were assessed the use of metformin and suggested an association with decreased mortality after a cancer diagnosis compared with diabetic and non- diabetic cancer patients (Xu et al., 2015).

Every single anti-diabetic drug does not show the similar tendency as an anti-cancer agent. The use of metformin was linked with reduced risk of PCa diagnosis, whereas other oral diabetic medications had no effect (Preston et al., 2014). Combination therapy was also another option to use metformin in cancer treatment. Hirsch et al. (2013) showed that combination of metformin with chemotherapy might obstruct tumor growth and prevent recurrence of PCa cells associated with blocking inflammatory pathways in xenograft models. Recent study showed that metformin and VPA synergistically repressed the proliferation in both LNCaP and PC-3. The combination therapy was also found to cause increased apoptosis in patient-derived prostate tumor explants. Concisely, two drugs have been suggested that working synergistically to destroy more PCa cells than either monotherapies alone (Tran et al., 2017).

### Zoledronic Acid

Zoledronic acid (ZA) is an aminobisphosphonate with high affinity for bone mineral and approved for the treatment of bone related diseases such as osteoporosis. When it is administered intravenously, it quickly localizes to bone and inhibits osteoclastic bone resorption by negative regulation of the action of the farnesyl pyrophosphate synthase enzyme in the mevalonate pathway (Lewiecki, 2009). Zoledronic acid is the first bisphosphonate with displayed efficacy in the treatment of bone metastases of patients with a broad range of tumor types including breast, prostate, and lung and in multiple myeloma as well as hypercalcemia of malignancy. It also reduces incidence of skeletal relevant complications and palliate the pain in patients with bone metastasis (Perry and Figgitt, 2004). In addition to other cancer types, there are more than 50 phase 4 clinical trials which are completed for PCa and bone metastasis according to https://clinicaltrials.gov.

The anti-cancer mechanisms of ZA may comprise apoptosis associated with the mevalonate pathway, activation of caspases, effects on invasiveness of the target cells, and arbitrated by changes in expression and activity of metalloproteinases (Corey et al., 2003). Considering PCa, ZA effectively prevents both bone loss in patients with locally advanced disease receiving androgen deprivation therapy and skeletal complications in men with hormone-refractory metastatic disease. Moreover, it assumed gold standard medication for the prevention and treatment of skeletal complications in patients with bone metastases due to all solid tumors with an acceptable safety profile and tolerability (Polascik and Mouraviev, 2008).

The ZA has known anti-PCa effects, demonstrated both preclinically with *in vitro* and *in vivo* studies (Corey et al., 2003) and clinically through placebo-controlled trials and long-term efficacy for the prevention of skeletal complications in later-stage disease (Saad et al., 2002, 2004). Combination therapy ZA with docetaxel demonstrated major improvements in survival of PCa patients (James et al., 2016), and recently, COX-2 inhibitors such as celecoxib and ZA therapy considered complementary for the treatment of advanced or metastatic PCa but findings showed no overall evidence of improved survival within the combinatorial therapy (Mason et al., 2017). ZA is generally used every 3 weeks based on initial clinical trials, however, the current evidences suggested similar outcomes with a reasonable biochemical response when ZA is used every 12 weeks in men with bone metastases from CRPC. Less frequent treatments may release the side effects, inconvenience, and cost (Niraula et al., 2018).

## Systems Biology Perspective in Drug Discovery for Prostate Cancer

**FIGURE 3:**
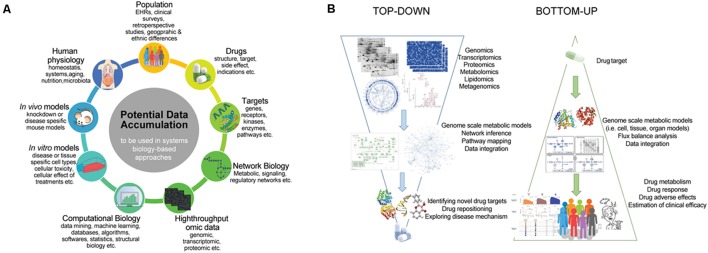
The use of system biology in the drug repositioning. **(A)** Potential data accumulation to be used in systems biology based approaches. **(B)** The application of two fundamental approaches in systems biology for drug design and discovery.

The preliminary drug targeting approaches were correlating diseases with the precise genetic malfunctions and subjected to discover only the drugs that modulate these gene targets. This reductionist point of view has not been totally successful since the biology behind diseases is complex and diseases are rarely up to single protein or gene (Schneider and Klabunde, 2013). In this manner, systems biology as a holistic approach can handle abundancy of high-throughput data from diverse omics techniques and other sources as well as their processes via mathematical modeling, and computational analysis tools (**Figure 3A**) (Mardinoglu and Nielsen, 2015; Zhang and Hua, 2016; Benfeitas et al., 2017; Mardinoglu et al., 2018a).

To date, systems biology approaches have been applied to decipher the disease mechanism and therapy options (Lee et al., 2016, 2017; Mardinoglu et al., 2017, 2018b). As an example, network biology approach called mode-of-action by network identification was used to identify the mediator genes and pathways in both primary and metastatic prostate tumors (Ergün et al., 2007). Besides, regulatory modular networks based on disease-specific miRNA-mRNA correlation were constructed to explore substantial variation across primary and metastatic prostate tumors (Zhang et al., 2012). Systems biology approaches also enable to discover potent biomarkers for predicting PCa outcomes as Ummanni et al. (2011) revealed novel protein biomarkers such as eIF4A3, DDAH1, ARG2, Prdx3, and Prdx4 associated with altered proteomic expression in PCa compared to benign prostate. There are also integrative studies combining multi-platform and multi-dimensional datasets. Comprehensive study utilizing different omics data from miRNA, mRNA, and protein expressions to copy number alterations was used for the effective stratification of patients with primary prostate tumor (Abeshouse et al., 2015). In another study, habitual genomics and transcriptomics data were integrated with phosphoproteomic to identify significant kinase signaling networks in advanced PCa (Tan et al., 2016).

The two fundamental approaches of systems biology, the “top-down” and “bottom-up” methods, are also applicable to the drug design and discovery in addition to other application such as network biology, biomarker discovery, etc. As pointed out in **Figure 3B**, top-down systems biology approaches aim to analyze high-throughput omics datasets to elucidate networks and logic behind the biological phenomena. From the drug discovery perspective, top-down methods provide detailed understanding of the causes of disease via perturbed networks and identifying reporter signatures as candidate biomarkers or drug targets. As an example of top-down methods, Jin et al. (2012) used transcriptional responses in PC-3 cells as well as MCF7 and HL60 before and after drug treatment. They developed drug off-target-based hybrid method that combines Bayesian factor regression model and network component called cancer-signaling bridges to analyze transcriptome data associated with drug usage. On the other hand, bottom-up methods object to simulate nature by utilizing known parameters and generating models of living cells. Each individual components of a system are gathered into a generated network to chase dynamic properties at the system level. Different computational modeling methods such as ODE-based, logic-based, or stoichiometric modeling can be applicable to investigate drug mechanism, its responses, and side effects. Gallaher et al. (2014) designed a computational model to predict patient outcome due to therapy response. They highlighted that such models and/or genetic algorithms are particularly useful for oncologists to predict the therapy response or drug combination therapies for effective treatment and cure of metastatic castrate resistant PCa patients.

Since the metabolic changes are quite important to detect and treat PCa regardless the disease stage (Eidelman et al., 2017), genome-scale metabolic models (GEMs) might be a very helpful source to create and/or test the hypothesis for drug repositioning in addition to elucidation of physiological mechanisms or side effects (Mardinoglu and Nielsen, 2012; Zhang and Hua, 2016) so GEMs can be used as a tool in both “top-down” and “bottom-up” methods in the context of drug discovery. GEMs have been employed for studying cancer metabolism utilizing either generic/personalized or tumor/cell-specific which may translate into clinically relevant applications. They can also be used to identify drug targets leading inhibition of cancer-related phenotypes or drug resistance in cancer therapy. Furthermore, the fortification of GEMs can be obtained via integration of omics data like genomic, transcriptomic, and proteomic as well as incorporation of regulatory molecules to the metabolism (Lee et al., 2018). GEMs also provide valuable insight on the interaction between cancer cells and supporting cells in their niches as paving the way for whole-cell modeling (Ghaffari et al., 2015; Yizhak et al., 2015). There are valuable GEMs studies for drug-related purposes. Metabolic transformation algorithm (MTA) was purposed to search for drug targets that could restore the metabolism within the cell from the disease state to the healthy state (Yizhak et al., 2013). Agren et al. (2014) revised the INIT algorithm to tINIT and used this for reconstruction of personalized GEMs for HCC patients and 83 healthy cell types via integration of proteomics data and HMR 2.0 (Mardinoglu et al., 2014). Reconstructed personalized GEMs were used to identify selective anti-metabolites for cancer therapy. GEMs were also used for predicting drug side effects and the identification of key metabolic reactions and biomarkers via implementation of model-based phenotype predictor approach that leverages medical informatics resources and GEMs (Shaked et al., 2016). PCa-specific generic GEM (Agren et al., 2012) and personalized GEMs for PCa patients (Uhlen et al., 2017) have been reconstructed for revealing the molecular mechanism of the tumor progression. They developed INIT algorithm and used HMR 2.0 to reconstruct cell type–specific metabolic models with information on protein abundances from the Human Proteome Atlas. Very recently, GEMs for two clonal subpopulations from PC-3 cell line were modeled to explore their metabolic differences. Highly invasive, low metastatic PC-3/S, and highly metastatic, low invasive PC-3/M cells present the opposite phenotype. Addition to unveiling key metabolic nodes related to tumor heterogeneity, results also highlighted potential subpopulation-specific targets with potential therapeutic implications. Such model for PC-3/M cells showed sensitivity to etomoxir, an inhibitor of long-chain fatty acid transport to the mitochondria (Marin de Mas et al., 2018).

## Future Directions

Known non-cancer drugs with new therapeutic applications in oncology has been reviewed in detail comprising drug mode of actions, rationale behind the approaches, practical evidences such as preclinical or clinical studies, and opportunities for repurposing drugs as anti-cancer agents (Gupta et al., 2013; Shim and Liu, 2014; Bhattarai et al., 2016; Würth et al., 2016).

Drugs from knowledge- and activity-based methods were quite more applied than *in silico* methods. Since the available drug libraries were already tested for PCa and there is still the need of expanded new drug libraries, these methods could be in bottleneck, however, *in silico* methods were not elaborated, yet but showed high potential for validation of drugs as well as discovery process which might be achieved via computational methods. Several studies have been discussing *in silico* methods and their integrations in detail (Jiao et al., 2015; Hodos et al., 2016; March-Vila et al., 2017; Vanhaelen et al., 2017).

Gut microbiota has a crucial role in drug efficacy both directly and indirectly. Hence, diet has also enormous influence on the gut microbiota, pioneering computational models comprising xenobiotic metabolism and microbial enzymes (Shoaie et al., 2015) should be constructed for integrating diet-microbiota-drug interactions and evaluated in the context of PCa treatment (Thiele et al., 2017).

Current standard treatment strategy for localized PCa consists of prostatectomy, RT supplemented with hormone therapy. However, recurrence after surgery or formation of castration-resistant PCa after hormone therapy is common. Chemotherapy has merely moderate effect for the treatment of metastatic tumor. Most of the drugs (repurposed or already used in clinic) target growth factors and/or their receptors, which are abundant in tumors. Some of them modulate survival, angiogenesis, or downstream signaling pathways. In general, researchers focused on metastatic PCa more than primary tumor because of high mortality and poor survival rates. However, primary prostate tumor with intermediate or high-risk score cause higher mortality regardless of initial therapy (Fu et al., 2012).

The most lethal form of PCa is considered as metastatic castration-resistant PCa whereas it is progressed form of primary tumors. Therefore, effective treatment strategies on prostate primary tumors are as important as on metastatic ones. The use of GEMs to detect metabolic alterations and their drug targets is already discussed in this review. However, there is a need for comprehensive model which presents not only metastatic PCa but also primary PCa. On the other hand, omics data integration is a significant value for a metabolic model in which generally transcriptomics and proteomics have utilized mainly. Whether GEMs are deliberated as generic disease–specific model, it is also important to represent each individual difference inside. Considering key stones, GEMs should be more evaluated for drug repositioning in the future.

Although DR is generally emphasized within advantages, many challenges deserve attention. Not all approved non-cancer drugs should be tested for cancer treatment without valid molecular insights into their potent efficacies. There are also several studies that take into account abandoned drugs for repurposing, however, extra care in selecting abandoned drugs is recommended regarding insufficient pharmacokinetic and pharmacodynamic data. Another point is uncertainties whether drug doses, formulations, and routes of administration for the original indication are similar to the need of the anti-cancer indication. Although approved drug acquired via DR methods do not work as expected, structures and targets of the approved drugs can also help in designing improved drug with new properties such as better water solubility, increased selectivity, long retention time, etc.

Although DR should significantly reduce the time and cost, considering the obstacles associated with phase 2 and 3 clinical trials, failure in these phases cannot be reduced by DR. Much money, larger number of patients and longer time is still needed after phase 2 of clinical trial with existing failure chance, unfortunately. Intellectual property right and regulatory standards comprising the discussions on off-patent drugs and their mechanism of action (MOU) patents are another issue considered for the commercialization of new cancer indication of an already approved drug as previously mentioned (Shim and Liu, 2014; Jiao et al., 2015; Bhattarai et al., 2016).

As we have mentioned in the study, North American studies reported a decreased risk of PCa (Vidal et al., 2015), whereas studies from Europe found elevated risk due to the use of any NSAID. Therefore, population and variations based on geographic region could be another challenge for DR. However, particularly *in silico* DR methodologies based on signature matching techniques might give expression signatures for each individual and expectedly translate into a personalized DR (Li and Jones, 2012; Hodos et al., 2016).

The DR provide significant opportunities to establish novel collaborations between academia and pharmaceutical industry. Moreover, there are some recent examples as mentioned previously. The National Institutes of Health has collaborated with pharmaceutical companies to investigate almost 60 abandoned drugs for new clinical purposes. Likewise, the United Kingdom Medical Research Council is making million dollars investments to the United Kingdom researchers for investigation of 22 abandoned compounds (Gupta et al., 2013).

## Conclusion

In this review, we highlighted repurposed non-cancer drugs and their mechanisms to treat PCa through diverse perspectives. Among these repurposed drugs, 10 drugs were obtained through different drug repositioning approaches as we categorized in 3: knowledge-based, activity-based, and *in silico* DR methods (**Table 1**). Most of them have shown anti-cancer activity only in preclinical studies. However, these observations will translate into the clinic remains to be seen. Another 14 drugs and 1 drug family (statins) were discussed in detail with a particular emphasis on non-cancer drugs under clinical investigation for PCa, up to the date (**Table 3**). Since drugs summarized in **Table 3** have already completed several phases of clinical trials, the known safety profiles of these drugs decrease their chances of failure to become an approved drug.

**FIGURE 4:**
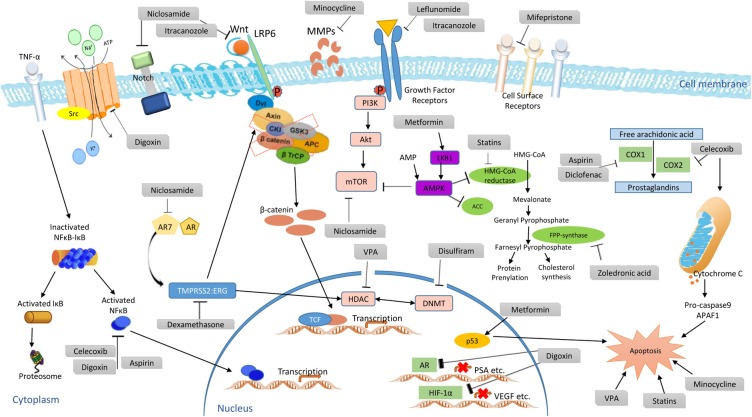
Proposed anti-cancer mechanisms of clinically evaluated repurposed drug candidates.

Mono- or combination therapy of these medications have shown anti-cancer and palliative activities in cancer patients. To the best of our knowledge, there are a few drugs such as thalidomide, celecoxib, methotrexate which have been approved for treatment of cancer patients, in addition to their original uses. However, zoledronic acid is the only repurposed and approved non-cancer drug for PCa patients this far. Anti-cancer activities of existing drugs shown in this study cover diverse mechanisms such as inhibition of mTOR and VEGFR2 signaling, PI3K/Akt signaling inhibition, COX and selective COX-2 inhibition, NF-κB inhibition, Wnt/β-Catenin pathway inhibition, DNMT1 inhibition, GSK-3β inhibition, suppression of anti-apoptotic proteins, tyrosine kinases and some growth factor inhibition, HIF-1α suppression, tumor angiogenesis reduction, and matrix metalloproteinases enzyme inhibition to resist invasion and metastasis (**Figure 4**).

Most of the repurposing studies summarized in this study were targeting metastatic or advanced PCa cases in which mortality rates were high and hormone therapy as far as medications do not help. However, treatment of primary tumors and prevention of the disease should also be considered in the context of drug repositioning. We anticipate that this review article will not only shed a light on current repurposed drugs but also help researchers in repurposing more therapeutics from already approved non-cancer drugs in the future.

## Author Contributions

BT wrote the manuscript. AM and KA conceived the study. All authors contributed to the manuscript during the progress of the work.

## Conflict of Interest Statement

The authors declare that the research was conducted in the absence of any commercial or financial relationships that could be construed as a potential conflict of interest.

## Abbreviations

AR: androgen receptor
CMT: chemically modified tetracycline
CRPC: castration-resistant prostate cancer
DR: drug repositioning
EMT: epithelial-to-mesenchymal transition
ER: endoplasmic reticulum
HDAC: histone deacetylases
HMG-CoA: 3-hydroxy-3-methylglutaryl
JHCCL: Johns Hopkins Clinical Compound Library
MMP: matrix metalloproteinases
NCGC: National Health Institute Chemical Genomics Center
NPC: NCGC pharmaceutical collection
NSAID: non-steroidal anti-inflammatory drug
PCa: prostate cancer
PSA: prostate-specific antigen
VPA: valproic acid
ZA: zoledronic acid

## References

[B1] Abeshouse A. Ahn J. Akbani R. Ally A. Amin S. Andry C. D. (2015). The molecular taxonomy of primary prostate cancer. 163 1011–1025. 10.1016/j.cell.2015.10.025 26544944 PMC4695400

[B2] Abou El-Magd R. Park H. Kawazoe T. Iwana S. Ono K. Chung S. (2010). The effect of risperidone on D-amino acid oxidase activity as a hypothesis for a novel mechanism of action in the treatment of schizophrenia. 24 1055–1067. 10.1177/0269881109102644 19329549

[B3] Aftab B. T. Dobromilskaya I. Liu J. O. Rudin C. M. (2011). Itraconazole inhibits angiogenesis and tumor growth in non-small cell lung cancer. 71 6764–6772. 10.1158/0008-5472.CAN-11-0691 21896639 PMC3206167

[B4] Agren R. Bordel S. Mardinoglu A. Pornputtapong N. Nookaew I. Nielsen J. (2012). Reconstruction of genome-scale active metabolic networks for 69 human cell types and 16 cancer types using INIT. 8:e1002518. 10.1371/journal.pcbi.1002518 22615553 PMC3355067

[B5] Agren R. Mardinoglu A. Asplund A. Kampf C. Uhlen M. Nielsen J. (2014). Identification of anticancer drugs for hepatocellular carcinoma through personalized genome-scale metabolic modeling. 10:721. 10.1002/msb.145122 24646661 PMC4017677

[B6] Antonarakis E. S. Heath E. I. Smith D. C. Rathkopf D. Blackford A. L. Danila D. C. (2013). Repurposing itraconazole as a treatment for advanced prostate cancer: a noncomparative randomized phase II trial in men with metastatic castration-resistant prostate cancer. 18 163–173. 10.1634/theoncologist.2012-314 23340005 PMC3579600

[B7] Assayag J. Pollak M. N. Azoulay L. (2015). The use of aspirin and the risk of mortality in patients with prostate cancer. 193 1220–1225. 10.1016/j.juro.2014.11.018 25463991

[B8] Ataie-Kachoie P. Morris D. L. Pourgholami M. H. (2013). Minocycline suppresses interleukine-6, its receptor system and signaling pathways and impairs migration, invasion and adhesion capacity of ovarian cancer cells: in vitro and in vivo studies. 8:e60817. 10.1371/journal.pone.0060817 23593315 PMC3620477

[B9] Ayan D. Maltais R. Poirier D. (2012). Identification of a 17β-hydroxysteroid dehydrogenase type10 steroidal inhibitor: a tool to investigate the role of type10 in Alzheimer’s disease and prostate cancer. 7 1181–1184. 10.1002/cmdc.201200129 22674910

[B10] Benfeitas R. Uhlen M. Nielsen J. Mardinoglu A. (2017). New challenges to study heterogeneity in cancer redox metabolism. 5:65. 10.3389/fcell.2017.00065 28744456 PMC5504267

[B11] Bhattarai D. Singh S. Jang Y. Han S. H. Lee K. Choi Y. (2016). An insight into drug repositioning for the development of novel anti-cancer drugs. 16 2156–2168. 10.2174/1568026616666160216153618 26881715

[B12] Bilani N. Bahmad H. Abou-Kheir W. (2017). Prostate cancer and aspirin use: Synopsis of the proposed molecular mechanisms. 8:145. 10.3389/fphar.2017.00145 28377721 PMC5359278

[B13] Breedveld F. C. Dayer J.-M. (2000). Leflunomide: mode of action in the treatment of rheumatoid arthritis. 59 841–849. 10.1136/ard.59.11.841 PMC1753034 11053058

[B14] Brodie S. A. Brandes J. C. (2014). Could valproic acid be an effective anticancer agent? The evidence so far. 14 1097–1100. 10.1586/14737140.2014.940329 25017212 PMC4579528

[B15] Buttgereit F. Burmester G. R. Straub R. H. Seibel M. J. Zhou H. (2011). Exogenous and endogenous glucocorticoids in rheumatic diseases. 63 1–9. 10.1002/art.30070 20936628

[B16] Calderón-Montaño J. Estefanía B.-M. Manuel Luis O. Maldonado-Navas D. (2014). Evaluating the cancer therapeutic potential of cardiac glycosides. 2014:794930. 10.1155/2014/794930 24895612 PMC4033509

[B17] Caon J. Paquette M. Hamm J. Pickles T. (2014). Does statin or ASA affect survival when prostate cancer is treated with external beam radiation therapy? 2014 184297. 10.1155/2014/184297 24729876 PMC3960556

[B18] Chan K. K. W. Oza A. M. Siu L. L. (2003). The statins as anticancer agents. 9 10–19. 10.1158/1078-0432.ccr-13-1524 12538446

[B19] Chang W.-L. Hsu L.-C. Leu W.-J. Chen C.-S. Guh J.-H. (2015). Repurposing of nitroxoline as a potential anticancer agent against human prostate cancer - a crucial role on AMPK/mTOR signaling pathway and the interplay with Chk2 activation. 6 39806–39820. 10.18632/oncotarget.5655 26447757 PMC4741862

[B20] Chong C. Sullivan D. J. (2007). New uses for old drugs. 448 645–646. 10.1038/448645a 17687303

[B21] Chong C. R. Chen X. Shi L. Liu J. O. Sullivan D. J. (2006a). A clinical drug library screen identifies astemizole as an antimalarial agent. 2 415–416. 10.1038/nchembio806 16816845

[B22] Chong C. R. Qian D. Z. Pan F. Wei Y. Pili R. Sullivan D. J. (2006b). Identification of type 1 inosine monophosphate dehydrogenase as an antiangiogenic drug target. 49 2677–2680. 10.1021/jm051225t 16640327

[B23] Chong C. R. Xu J. Lu J. Bhat S. Sullivan D. J. Liu J. O. (2007). Inhibition of angiogenesis by the antifungal drug itraconazole. 2 263–270. 10.1021/cb600362d 17432820

[B24] Chu C. C. Hsing C. H. Shieh J. P. Chien C. C. Ho C. M. Wang J. J. (2014). The cellular mechanisms of the antiemetic action of dexamethasone and related glucocorticoids against vomiting. 722 48–54. 10.1016/j.ejphar.2013.10.008 24184695

[B25] Corey E. Brown L. G. Quinn J. E. Poot M. Roudier M. P. Higano C. S. (2003). Zoledronic acid exhibits inhibitory effects on osteoblastic and osteolytic metastases of prostate cancer. 9 295–306. 12538482

[B26] Davies N. M. McLachlan A. J. Day R. O. Williams K. M. (2000). Clinical pharmacokinetics and pharmacodynamics of celecoxib: a selective cyclo-oxygenase-2 inhibitor. 38 225–242. 10.2165/00003088-200038030-00003 10749518

[B27] Deotarse P. P. Jain A. S. Baile M. B. Kolhe N. S. Kulkarni A. A. (2015). Drug repositioning: a review. 4 51–58.

[B28] Dilly S. J. Clark A. J. Marsh A. Mitchell D. A. Cain R. Fishwick C. W. G. (2017). A chemical genomics approach to drug reprofiling in oncology: antipsychotic drug risperidone as a potential adenocarcinoma treatment. 393 16–21. 10.1016/j.canlet.2017.01.042 28188816

[B29] Downer M. K. Allard C. B. Preston M. A. Gaziano J. M. Stampfer M. J. Mucci L. A. (2017). Regular aspirin use and the risk of lethal prostate cancer in the physicians’ Health Study. 72 821–827. 10.1016/j.eururo.2017.01.044 28189429

[B30] Eidelman E. Twum-Ampofo J. Ansari J. Siddiqui M. M. (2017). The metabolic phenotype of prostate cancer. 7:131. 10.3389/fonc.2017.00131 28674679 PMC5474672

[B31] El Etreby M. F. Liang Y. Lewis R. W. (2000). Induction of apoptosis by mifepristone and tamoxifen in human LNCaP prostate cancer cells in culture. 43 31–42. 10.1002/(SICI)1097-0045(20000401)43:1<31::AID-PROS5>3.0.CO;2-10725863

[B32] Elder D. J. E. Halton D. E. Crew T. E. Paraskeva C. (2000). Apoptosis induction and cyclooxygenase-2 regulation in human colorectal adenoma and carcinoma cell lines by the cyclooxygenase-2-selective non-steroidal anti-inflammatory drug Ns-398. 86 553–560. 10.1002/(SICI)1097-0215(20000515)86:4<553::AID-IJC18>3.0.CO;2-9 10797271

[B33] Ergün A. Lawrence C. A. Kohanski M. A. Brennan T. A. Collins J. J. (2007). A network biology approach to prostate cancer. 3:82. 10.1038/msb4100125 17299418 PMC1828752

[B34] Evans J. M. M. Donnelly L. A. Emslie-Smith A. M. Alessi D. R. Morris A. D. (2005). Metformin and reduced risk of cancer in diabetic patients. 330 1304–1305. 10.1136/bmj.38415.708634.F7 15849206 PMC558205

[B35] Ferlay J. Soerjomataram I. Dikshit R. Eser S. Mathers C. Rebelo M. (2015). Cancer incidence and mortality worldwide: Sources, methods and major patterns in GLOBOCAN 2012. 136 E359–E386. 10.1002/ijc.29210 25220842

[B36] Fowke J. H. Motley S. S. Smith J. A. Cookson M. S. Concepcion R. Chang S. S. (2009). Association of nonsteroidal anti-inflammatory drugs, prostate specific antigen and prostate volume. 181 2064–2070. 10.1016/j.juro.2009.01.031 19286210 PMC2679527

[B37] Fu W. Madan E. Yee M. Zhang H. (2012). Progress of molecular targeted therapies for prostate cancers. 1825 140–152. 10.1016/j.bbcan.2011.11.003 22146293 PMC3307854

[B38] Gallaher J. Cook L. M. Gupta S. Araujo A. Dhillon J. Park J. Y. (2014). Improving treatment strategies for patients with metastatic castrate resistant prostate cancer through personalized computational modeling. 31 991–999. 10.1007/s10585-014-9674-1 25173680 PMC5399888

[B39] Gantt S. Casper C. Ambinder R. F. (2013). Insights into the broad cellular effects of nelfinavir and the HIV protease inhibitors supporting their role in cancer treatment and prevention. 25 495–502. 10.1097/CCO.0b013e328363dfee 23872785 PMC4029099

[B40] Garrido-Mesa N. Zarzuelo A. Gálvez J. (2013). Minocycline: Far beyond an antibiotic. 169 337–352. 10.1111/bph.12139 23441623 PMC3651660

[B41] Gayvert K. M. Dardenne E. Cheung C. Boland M. R. Lorberbaum T. Wanjala J. (2016). A computational drug repositioning approach for targeting oncogenic transcription factors. 15 2348–2356. 10.1016/j.celrep.2016.05.037 27264179 PMC4912004

[B42] Ghaffari P. Mardinoglu A. Asplund A. Shoaie S. Kampf C. Uhlen M. (2015). Identifying anti-growth factors for human cancer cell lines through genome-scale metabolic modeling. 5:8183. 10.1038/srep08183 25640694 PMC4313100

[B43] Giridhar R. (2012). Drug discovery: past and present. 3:2. 10.4103/2231-4040.93554 PMC3312722 22470886

[B44] Gonnissen A. Isebaert S. McKee C. M. Dok R. Haustermans K. Muschel R. J. (2016). The hedgehog inhibitor GANT61 sensitizes prostate cancer cells to ionizing radiation both *in vitro* and *in vivo*. 7 84286–84298. 10.18632/oncotarget.12483 27713179 PMC5356662

[B45] Gottfried E. Lang S. A. Renner K. Bosserhoff A. Gronwald W. Rehli M. (2013). New aspects of an old drug - diclofenac targets MYC and glucose metabolism in tumor cells. 8:e66987. 10.1371/journal.pone.0066987 23874405 PMC3706586

[B46] Grösch S. Maier T. J. Schiffmann S. Geisslinger G. (2006). Cyclooxygenase-2 (COX-2)-independent anticarcinogenic effects of selective COX-2 inhibitors. 98 736–747. 10.1093/jnci/djj206 16757698

[B47] Guan M. Su L. Yuan Y. C. Li H. Chow W. A. (2015). Nelfinavir and nelfinavir analogs block site-2 protease cleavage to inhibit castration-resistant prostate cancer. 5:9698. 10.1038/srep09698 25880275 PMC4816264

[B48] Gupta S. C. Sung B. Prasad S. Webb L. J. Aggarwal B. B. (2013). Cancer drug discovery by repurposing: Teaching new tricks to old dogs. 34 508–517. 10.1016/j.tips.2013.06.005 23928289

[B49] Hafeez B. B. Ganju A. Sikander M. Kashyap V. K. Hafeez Z. Bin (2017). Ormeloxifene suppresses prostate tumor growth and metastatic phenotypes via inhibition of oncogenic β-catenin signaling and EMT progression. 16 2267–2280. 10.1158/1535-7163.MCT-17-0157 28615299 PMC5774234

[B50] Hail N. Chen P. Bushman L. R. (2010). Teriflunomide (Leflunomide) promotes cytostatic, antioxidant, and apoptotic effects in transformed prostate epithelial cells: evidence supporting a role for teriflunomide in prostate cancer chemoprevention. 12 464–475. 10.1593/neo.10168 20563249 PMC2887087

[B51] Harris R. E. Alshafie G. A. Abou-Issa H. Seibert K. (2000). Chemoprevention of breast cancer in rats by celecoxib, a cyclooxygenase 2 inhibitor. 60 2101–2103. 10786667

[B52] Hazra B. G. Pore V. S. (2001). Mifepristone (RU-486), the recently developed antiprogesterone drug and its analogues. 81 287–298. 3069265

[B53] Hindler K. (2006). The role of statins in cancer therapy. 11 306–315. 10.1634/theoncologist.11-3-306 16549815

[B54] Hirsch H. A. Iliopoulos D. Struhl K. (2013). Metformin inhibits the inflammatory response associated with cellular transformation and cancer stem cell growth. 110 972–977. 10.1073/pnas.1221055110 23277563 PMC3549132

[B55] Hodos R. A. Kidd B. A. Khader S. Readhead B. P. Dudley J. T. (2016). In silico methods for drug repurposing and pharmacology. 8 186–210. 10.1002/wsbm.1337 27080087 PMC4845762

[B56] Hsu A. L. Ching T. T. Wang D. S. Song X. Rangnekar V. M. Chen C. S. (2000). The cyclooxygenase-2 inhibitor celecoxib induces apoptosis by blocking Akt activation in human prostate cancer cells independently of Bcl-2. 275 11397–11403. 10.1074/jbc.275.15.11397 10753955

[B57] Huang R. Southall N. Wang Y. Yasgar A. Shinn P. Jadhav A. (2011). The NCGC pharmaceutical collection: a comprehensive resource of clinically approved drugs enabling repurposing and chemical genomics. 3:80ps16. 10.1126/scitranslmed.3001862 21525397 PMC3098042

[B58] Hutchinson J. Marignol L. (2017). Clinical potential of statins in prostate cancer radiation therapy. 37 5363–5372. 10.21873/anticanres.11962 28982844

[B59] Iljin K. Ketola K. Vainio P. Halonen P. Kohonen P. Fey V. (2009). High-throughput cell-based screening of 4910 known drugs and drug-like small molecules identifies disulfiram as an inhibitor of prostate cancer cell growth. 15 6070–6078. 10.1158/1078-0432.CCR-09-1035 19789329

[B60] Inoue T. Anai S. Onishi S. Miyake M. Tanaka N. Hirayama A. (2013). Inhibition of COX-2 expression by topical diclofenac enhanced radiation sensitivity via enhancement of TRAIL in human prostate adenocarcinoma xenograft model. 13:1. 10.1186/1471-2490-13-1 23289871 PMC3561196

[B61] Iwamoto Y. Ishii K. Kanda H. Kato M. Miki M. Kajiwara S. (2017). Combination treatment with naftopidil increases the efficacy of radiotherapy in PC-3 human prostate cancer cells. 143 933–939. 10.1007/s00432-017-2367-9 28243746 PMC11818994

[B62] Jaggi M. Johansson S. L. Baker J. J. Smith L. M. Galich A. Balaji K. C. (2005). Aberrant expression of E-cadherin and beta-catenin in human prostate cancer. 23 402–406. 10.1016/j.urolonc.2005.03.024 16301117

[B63] Jaggi M. Nazemi T. Abrahams N. A. Baker J. J. Galich A. Smith L. M. (2006). N-cadherin switching occurs in high Gleason grade prostate cancer. 66 193–199. 10.1002/pros.20334 16173043

[B64] Jalving M. Gietema J. A. Lefrandt J. D. de Jong S. Reyners A. K. L. Gans R. O. B. (2010). Metformin: Taking away the candy for cancer? 46 2369–2380. 10.1016/j.ejca.2010.06.012 20656475

[B65] James N. D. Sydes M. R. Clarke N. W. Mason M. D. Dearnaley D. P. Spears M. R. (2016). Addition of docetaxel, zoledronic acid, or both to first-line long-term hormone therapy in prostate cancer (STAMPEDE): survival results from an adaptive, multiarm, multistage, platform randomised controlled trial. 387 1163–1177. 10.1016/S0140-6736(15)01037-5 PMC4800035 26719232

[B66] Jiang H. Taggart J. E. Zhang X. Benbrook D. M. Lind S. E. Ding W.-Q. (2012). Nitroxoline (5-amino-8-hydroxyquinoline) is more a potent anticancer agent than clioquinol (5-chloro-7-iodo-8-quinoline). 312 11–17. 10.1016/j.canlet.2011.06.032.Nitroxoline PMC3395224 21899946

[B67] Jiao M. Liu G. Xue Y. Ding C. (2015). Computational drug repositioning for cancer therapeutics. 15 767–775. 10.2174/1568026615666150302105831 25732789

[B68] Jin G. Fu C. Zhao H. Cui K. Chang J. Wong S. T. C. (2012). A novel method of transcriptional response analysis to facilitate drug repositioning for cancer therapy. 72 33–44. 10.1158/0008-5472.CAN-11-2333 22108825 PMC3251651

[B69] Kalra G. de Sousa A. Shrivastava A. (2014). Disulfiram in the management of alcohol dependence: a comprehensive clinical review. 4 43–52. 10.1111/nyas.12538 25236185 PMC4206699

[B70] Kar A. (2007). New Delhi: New age International Publishers.

[B71] Katz M. S. Carroll P. R. Cowan J. E. Chan J. M. D’Amico A. V. (2010). Association of statin and nonsteroidal anti-inflammatory drug use with prostate cancer outcomes: results from CaPSURE. 106 627–632. 10.1111/j.1464-410X.2010.09232.x 20151961

[B72] Kim J. Tang J. Y. Gong R. Kim J. Lee J. J. Clemons K. V. (2010). Itraconazole, a commonly used antifungal that inhibits hedgehog pathway activity and cancer growth. 17 388–399. 10.1016/j.ccr.2010.02.027 20385363 PMC4039177

[B73] Kirschenbaum A. Liu X. H. Yao S. Levine A. C. (2001). The role of cyclooxygenase-2 in prostate cancer. 58(2 Suppl. 1), 127–131. 10.1016/S0090-4295(01)01255-9 11502467

[B74] Ko Y. J. Small E. J. Kabbinavar F. Chachoua A. Taneja S. Reese D. (2001). A multi-institutional phase ii study of SU101, a platelet-derived growth factor receptor inhibitor, for patients with hormone-refractory prostate cancer. 7 800–805. 11309325

[B75] Koltai T. (2015). Fenofibrate in cancer: mechanisms involved in anticancer activity. 4:55 10.12688/f1000research.6153.2 PMC4457118 26097685

[B76] Kondratskyi A. Kondratska K. Vanden Abeele F. Gordienko D. Dubois C. Toillon R. A. (2017). Ferroquine, the next generation antimalarial drug, has antitumor activity. 7:15896. 10.1038/s41598-017-16154-2 29162859 PMC5698296

[B77] Kulp S. K. Yang Y. Hung C. Chen K. Lai J. Tseng P. (2004). 3-Phosphoinositide-dependent protein kinase-1 / Akt signaling represents a major cyclooxygenase-2-independent target for celecoxib in prostate cancer cells 3-phosphoinositide-dependent protein kinase-1 / Akt signaling represents a major cyclooxygenase-2-I. 64 1444–1451. 10.1158/0008-5472.CAN-03-2396 14973075

[B78] Leahy K. M. Ornberg R. L. Wang Y. Zweifel B. S. Koki A. T. Masferrer J. L. (2002). Cyclooxygenase-2 inhibition by celecoxib reduces proliferation and induces apoptosis in angiogenic endothelial cells in vivo. 62 625–631. 11830509

[B79] Lee J. E. Kim J. H. (2015). Valproic acid inhibits the invasion of PC3 prostate cancer cells by upregulating the metastasis suppressor protein NDRG1. 38 527–533. 10.1590/S1415-475738420150028 26692161 PMC4763324

[B80] Lee S. Zhang C. Arif M. Liu Z. Benfeitas R. Bidkhori G. (2018). TCSBN: A database of tissue and cancer specific biological networks. 46 D595–D600. 10.1093/nar/gkx994 29069445 PMC5753183

[B81] Lee S. Zhang C. Kilicarslan M. Piening B. D. Bjornson E. Hallström B. M. (2016). Integrated network analysis reveals an association between plasma mannose levels and insulin resistance. 24 172–184. 10.1016/j.cmet.2016.05.026 27345421 PMC6666317

[B82] Lee S. Zhang C. Liu Z. Klevstig M. Mukhopadhyay B. Bergentall M. (2017). Network analyses identify liver-specific targets for treating liver diseases. 13:938. 10.15252/msb.20177703 28827398 PMC5572395

[B83] Levy C. W. Roujeinikova A. Sedelnikova S. Baker P. J. Stuitje A. R. Slabas A. R. (1999). Molecular basis of triclosan activity. 398 383–384. 10.1038/18803 10201369

[B84] Lewiecki E. M. (2009). Intravenous zoledronic acid for the treatment of osteoporosis: the evidence of its therapeutic effect. 4 13–23. 10.2147/CE.S6011 PMC2899787 20694061

[B85] Li Y. Y. Jones S. J. (2012). Drug repositioning for personalized medicine. 4:27. 10.1186/gm326 22494857 PMC3446277

[B86] Lin J. Haffner M. C. Zhang Y. Lee B. H. Brennen W. N. Britton J. (2011). Disulfiram is a DNA demethylating agent and inhibits prostate cancer cell growth. 71 333–343. 10.1002/pros.21247 20809552 PMC3043358

[B87] Lin J. Zhan T. Duffy D. Hoffman-Censits J. Kilpatrick D. Trabulsi E. J. (2014). A pilot phase II study of digoxin in patients with recurrent prostate cancer as evident by a rising PSA. 2 21–32. 25580468 PMC4287984

[B88] Liu Y. Chen J. Q. Xie L. Wang J. Li T. He Y. (2014). Effect of aspirin and other non-steroidal anti-inflammatory drugs on prostate cancer incidence and mortality: a systematic review and meta-analysis. 12:55. 10.1186/1741-7015-12-55 24678716 PMC4021622

[B89] Lokeshwar B. L. (2011). Chemically modified non-antimicrobial tetracyclines are multifunctional drugs against advanced cancers. 63 146–150. 10.1016/j.phrs.2010.11.003 21093590 PMC3031750

[B90] Lopez-Lazaro M. (2009). Digoxin, HIF-1, and cancer. 106:E26. 10.1073/pnas.0813047106 19240208 PMC2651277

[B91] Lu W. Lin C. Roberts M. J. Waud W. R. Piazza G. A. Li Y. (2011). Niclosamide suppresses cancer cell growth by inducing Wnt co-receptor LRP6 degradation and inhibiting the Wnt/β-catenin pathway. 6:e29290. 10.1371/journal.pone.0029290 22195040 PMC3241710

[B92] Mahmud S. M. Franco E. L. Aprikian A. G. (2010). Use of nonsteroidal anti-inflammatory drugs and prostate cancer risk: a meta-analysis. 127 1680–1691. 10.1002/ijc.25186 20091856

[B93] Marcella S. W. David A. Ohman-Strickland P. A. Carson J. Rhoads G. G. (2012). Statin use and fatal prostate cancer: a matched case-control study. 118 4046–4052. 10.1002/cncr.26720 22180145 PMC4314096

[B94] March-Vila E. Pinzi L. Sturm N. Tinivella A. Engkvist O. Chen H. (2017). On the integration of in silico drug design methods for drug repurposing. 8:298 10.3389/fphar.2017.00298 PMC5440551 28588497

[B95] Mardinoglu A. Agren R. Kampf C. Asplund A. Uhlen M. Nielsen J. (2014). Genome-scale metabolic modelling of hepatocytes reveals serine deficiency in patients with non-alcoholic fatty liver disease. 5:3083. 10.1038/ncomms4083 24419221

[B96] Mardinoglu A. Bjornson E. Zhang C. Klevstig M. Söderlund S. Ståhlman M. (2017). Personal model-assisted identification of NAD + and glutathione metabolism as intervention target in NAFLD. 13:916. 10.15252/msb.20167422 28254760 PMC5371732

[B97] Mardinoglu A. Boren J. Smith U. Uhlen M. Nielsen J. (2018a). Systems biology in hepatology: approaches and applications. (in press). 10.1038/s41575-018-0007-8 29686404

[B98] Mardinoglu A. Nielsen J. (2012). Systems medicine and metabolic modelling. 271 142–154. 10.1111/j.1365-2796.2011.02493.x 22142312

[B99] Mardinoglu A. Nielsen J. (2015). New paradigms for metabolic modeling of human cells. 34 91–97. 10.1016/j.copbio.2014.12.013 25559199

[B100] Mardinoglu A. Wu H. Bjornson E. Zhang C. Hakkarainen A. Räsänen S. M. (2018b). An integrated understanding of the rapid metabolic benefits of a carbohydrate-restricted diet on hepatic steatosis in humans. 27 559.e5–571.e5. 10.1016/j.cmet.2018.01.005 29456073 PMC6706084

[B101] Margel D. Urbach D. R. Lipscombe L. L. Bell C. M. Kulkarni G. Austin P. C. (2013). Metformin use and all-cause and prostate cancer-specific mortality among men with diabetes. 31 3069–3075. 10.1200/JCO.2012.46.7043 23918942

[B102] Marin de Mas I. Aguilar E. Zodda E. Balcells C. Marin S. Dallmann G. (2018). Model-driven discovery of long-chain fatty acid metabolic reprogramming in heterogeneous prostate cancer cells. 14:e1005914. 10.1371/journal.pcbi.1005914 29293497 PMC5766231

[B103] Mason M. D. Clarke N. W. James N. D. Dearnaley D. P. Spears M. R. Ritchie A. W. S. (2017). Adding celecoxib with or without zoledronic acid for hormone-naïve prostate cancer: long-term survival results from an adaptive, multiarm, multistage, platform, randomized controlled trial. 35 1530–1541. 10.1200/JCO.2016.69.0677 28300506 PMC5455701

[B104] Masumori N. (2011). Naftopidil for the treatment of urinary symptoms in patients with benign prostatic hyperplasia. 7 227–238. 10.2147/TCRM.S13883 21753885 PMC3132093

[B105] McMahon M. A. Jilek B. L. Brennan T. P. Shen L. Zhou Y. Wind-Rotolo M. (2007). The HBV drug entecavir — Effects on HIV-1 replication and resistance. 356 2614–2621. 10.1056/NEJMoa067710 17582071 PMC3069686

[B106] McMurry L. M. Oethinger M. Levy S. B. (1998). Triclosan targets lipid synthesis. 394 531–532. 10.1038/28970 9707111

[B107] Michaelis M. Doerr H. W. Cinatl J. (2007). Valproic acid as anti-cancer drug. 13 3378–3393. 10.2174/138161207782360519 18045192

[B108] Mobbs B. G. Johnson I. E. (1991). Suppression of the growth of the androgen-insensitive R3327 hi rat prostatic carcinoma by combined estrogen and antiprogestin treatment. 39 713–722. 10.1016/0960-0760(91)90371-B 1958508

[B109] Niraula S. Templeton A. J. Vera-Badillo F. Dodd A. Nugent Z. Joshua A. M. (2018). Duration of suppression of bone turnover following treatment with zoledronic acid in men with metastatic castration-resistant prostate cancer. 4:FSO253. 10.4155/fsoa-2017 29255625 PMC5729592

[B110] Noto H. Goto A. Tsujimoto T. Noda M. (2012). Cancer risk in diabetic patients treated with metformin: a systematic review and meta-analysis. 7:e33411. 10.1371/journal.pone.0033411 22448244 PMC3308971

[B111] Padhy B. M. Gupta Y. K. (2011). Drug repositioning: re-investigating existing drugs for new therapeutic indications. 57 153–160. 10.4103/0022-3859.81870 21654146

[B112] Pan J. X. Ding K. Wang C. Y. (2012). Niclosamide, an old antihelminthic agent, demonstrates antitumor activity by blocking multiple signaling pathways of cancer stem cells. 31 178–184. 10.5732/cjc.011.10290 22237038 PMC3777479

[B113] Pantziarka P. Bouche G. Meheus L. Sukhatme V. Sukhatme V. P. (2014). The repurposing drugs in oncology (ReDO) project. 8:442 10.3332/ecancer.2014.442 PMC4096030 25075216

[B114] Pantziarka P. Sukhatme V. Bouche G. Meheus L. Sukhatme V. P. (2015). Repurposing drugs in oncology (ReDO)—itraconazole as an anti-cancer agent. 9:521 10.3332/ecancer.2015.521 PMC4406527 25932045

[B115] Pantziarka P. Sukhatme V. V. P. Bouche G. Melhuis L. Sukhatme V. V. P. (2016). Repurposing drugs in oncology (ReDO)—diclofenac as an anti-cancer agent. 10:610. 10.3332/ecancer.2016.610 26823679 PMC4720497

[B116] Patel M. I. Subbaramaiah K. Du B. Patel M. I. Subbaramaiah K. Du B. (2005). Celecoxib inhibits prostate cancer growth: evidence of a cyclooxygenase-2-independent mechanism. 11 1999–2007. 10.1158/1078-0432.CCR-04-1877 15756026

[B117] Pelletier J. Bordeleau M. Lindqvist L. Francis R. Sukarieh R. Tanaka J. (2007). Chemotherapeutic agents for inhibition of protein translation Vol. WO2007/009264 A1 A61K 31/58 Edn.

[B118] Perry C. M. Figgitt D. P. (2004). Zoledronic acid: a review of its use in patients with advanced cancer. 64 1197–1211. 10.2165/00003495-200464110-00004 15161327

[B119] Piao S. Amaravadi R. K. (2016). Targeting the lysosome in cancer. 1371 45–54. 10.1111/nyas.12953 26599426 PMC4879098

[B120] Platz E. A. Yegnasubramanian S. Liu J. O. Chong C. R. Shim J. S. Kenfield S. A. (2011). A novel two-stage, transdisciplinary study identifies digoxin as a possible drug for prostate cancer treatment. 1 68–77. 10.1158/2159-8274.CD-100020 PMC3227223 22140654

[B121] Polascik T. J. Mouraviev V. (2008). Zoledronic acid in the management of metastatic bone disease. 4 261–268. 10.2147/TCRM.S2707 PMC2503661 18728715

[B122] Ponder B. (1997). Genetic testing for cancer risk. 278 1050–1054. 10.1126/science.278.5340.1050 9353178

[B123] Preston M. A. Riis A. H. Ehrenstein V. Breau R. H. Batista J. L. Olumi A. F. (2014). Metformin use and prostate cancer risk. 66 1012–1020. 10.1016/j.eururo.2014.04.027 24857538

[B124] Qi C. Li B. Yang Y. Y. Yang Y. Y. Li J. J. Zhou Q. (2016). Glipizide suppresses prostate cancer progression in the TRAMP model by inhibiting angiogenesis. 6:27819. 10.1038/srep27819 27292155 PMC4904209

[B125] Rebecca V. W. Amaravadi R. K. (2016). Emerging strategies to effectively target autophagy in cancer. 35 1–11. 10.1038/onc.2015.99 25893285 PMC4838040

[B126] Regen F. Heuser I. Herzog I. Hellmann-Regen J. (2014). Striking growth-inhibitory effects of minocycline on human prostate cancer cell lines. 83 509.e1–506.e1. 10.1016/j.urology.2013.10.029 24360070

[B127] Roth B. L. Sheffler D. J. Kroeze W. K. (2004). Magic shotguns versus magic bullets: selectively non-selective drugs for mood disorders and schizophrenia. 3 353–359. 10.1038/nrd1346 15060530

[B128] Rudin C. M. Brahmer J. R. Juergens R. A. Hann C. L. Ettinger D. S. Sebree R. (2013). Phase 2 study of pemetrexed and itraconazole as second-line therapy for metastatic nonsquamous non-small-cell lung cancer. 8 619–623. 10.1097/JTO.0b013e31828c3950 23546045 PMC3636564

[B129] Saad F. Gleason D. M. Murray R. Tchekmedyian S. Venner P. Lacombe L. (2002). A randomized, placebo-controlled trial of zoledronic acid in patients with hormone-refractory metastatic prostate carcinoma. 94 1458–1468. 10.1093/jnci/94.19.1458 12359855

[B130] Saad F. Gleason D. M. Murray R. Tchekmedyian S. Venner P. Lacombe L. (2004). Long-term efficacy of zoledronic acid for the prevention of skeletal complications in patients with metastatic hormone-refractory prostate cancer. 96 879–882. 10.1093/jnci/djh141 15173273

[B131] Sack U. Walther W. Scudiero D. Selby M. Kobelt D. Lemm M. (2011). Novel effect of antihelminthic niclosamide on s100a4-mediated metastatic progression in colon cancer. 103 1018–1036. 10.1093/jnci/djr190 21685359

[B132] Sadowski M. C. Pouwer R. H. Gunter J. H. Lubik A. A. Quinn R. J. Nelson C. C. (2014). The fatty acid synthase inhibitor triclosan: repurposing an anti- microbial agent for targeting prostate cancer. 5 9362–9381. 10.18632/oncotarget.2433 25313139 PMC4253440

[B133] Safi R. Nelson E. R. Chitneni S. K. Franz K. J. George D. J. Zalutsky M. R. (2014). Copper signaling axis as a target for prostate cancer therapeutics. 74 5819–5831. 10.1158/0008-5472.CAN-13-3527 25320179 PMC4203427

[B134] Schemmel K. E. Padiyara R. S. D’Souza J. J. (2010). Aldose reductase inhibitors in the treatment of diabetic peripheral neuropathy: a review. 24 354–360. 10.1016/j.jdiacomp.2009.07.005 19748287

[B135] Schneider H. C. Klabunde T. (2013). Understanding drugs and diseases by systems biology? 23 1168–1176. 10.1016/j.bmcl.2012.12.031 23337596

[B136] Schweizer M. T. Lin J. Blackford A. Bardia A. King S. Armstrong A. J. (2013). Pharmacodynamic study of disulfiram in men with non-metastatic recurrent prostate cancer. 16 357–361. 10.1038/pcan.2013.28 23958896 PMC3830644

[B137] Shaked I. Oberhardt M. A. Atias N. Sharan R. Ruppin E. (2016). Metabolic network prediction of drug side effects. 2 209–213. 10.1016/j.cels.2016.03.001 27135366

[B138] Shamash J. Powles T. Sarker S. J. Protheroe A. Mithal N. Mills R. (2011). A multi-centre randomised phase III trial of Dexamethasone vs Dexamethasone and diethylstilbestrol in castration-resistant prostate cancer: immediate vs deferred Diethylstilbestrol. 104 620–628. 10.1038/bjc.2011.7 21285990 PMC3049603

[B139] Sharma S. Symanowski J. Wong B. Dino P. Manno P. Vogelzang N. (2008). A phase II clinical trial of oral valproic acid in patients with castration-resistant prostate cancers using an intensive biomarker sampling strategy. 1 141–147. 10.1593/tlo.08136 18795124 PMC2533142

[B140] Shawver K. Schwartz P. Mann E. Chen H. Tsai J. Chu L. (1997). Inhibition of platelet-derived growth factor-mediated signal transduction and tumor growth by N-[4-(trifluoromethyl)-phenyl]5-methylisoxazole-4-carboxamide. 3 1167–1177 9815796

[B141] Shim J. S. Liu J. O. (2014). Recent advances in drug repositioning for the discovery of new anticancer drugs. 10 654–663. 10.7150/ijbs.9224 25013375 PMC4081601

[B142] Shim J. S. Matsui Y. Bhat S. Nacev B. A. Xu J. Bhang H. E. C. (2010). Effect of nitroxoline on angiogenesis and growth of human bladder cancer. 102 1855–1873. 10.1093/jnci/djq457 21088277 PMC3001967

[B143] Shoaie S. Ghaffari P. Kovatcheva-Datchary P. Mardinoglu A. Sen P. Pujos-Guillot E. (2015). Quantifying diet-induced metabolic changes of the human gut microbiome. 22 320–331. 10.1016/j.cmet.2015.07.001 26244934

[B144] Siegel R. L. Miller K. D. Jemal A. (2018). Cancer statistics, 2018. 68 7–30. 10.3322/caac.21442 29313949

[B145] Sirota M. Dudley J. T. Kim J. Chiang A. P. Morgan A. A. Sweet-Cordero A. (2011). Discovery and preclinical validation of drug indications using compendia of public gene expression data. 77:96ra77. 10.1126/scitranslmed.3001318 21849665 PMC3502016

[B146] Skrott Z. Mistrik M. Andersen K. K. Friis S. Majera D. Gursky J. (2017). Alcohol-abuse drug disulfiram targets cancer via p97 segregase adaptor NPL4. 552 194–199. 10.1038/nature25016 29211715 PMC5730499

[B147] Slingerland M. Cerella C. Guchelaar H. J. Diederich M. Gelderblom H. (2013). Cardiac glycosides in cancer therapy: From preclinical investigations towards clinical trials. 31 1087–1094. 10.1007/s10637-013-9984-1 23748872

[B148] Sosiè I. Mirkoviæ B. Arenz K. Štefane B. Kos J. Gobec S. (2013). Development of new cathepsin b inhibitors: combining bioisosteric replacements and structure-based design to explore the structure-activity relationships of nitroxoline derivatives. 56 521–533. 10.1021/jm301544x 23252745

[B149] Sun P. Guo J. Winnenburg R. Baumbach J. (2017). Drug repurposing by integrated literature mining and drug–gene–disease triangulation. 22 615–619. 10.1016/j.drudis.2016.10.008 27780789

[B150] Sussman G. L. Mason J. Compton D. Stewart J. Ricard N. (1999). The efficacy and safety of fexofenadine HCl and pseudoephedrine, alone and in combination, in seasonal allergic rhinitis. 104 100–106. 10.1016/S0091-6749(99)70120-X 10400846

[B151] Tammali R. Srivastava S. K. Ramana K. V. (2011). Targeting aldose reductase for the treatment of cancer. 11 560–571. 10.2174/156800911795655958 PMC3142792 21486217

[B152] Tan N. Klein E. A. Li J. Moussa A. S. Jones J. S. (2011). Statin use and risk of prostate cancer in a population of men who underwent biopsy. 186 86–90. 10.1016/j.juro.2011.03.004 21571344

[B153] Tan X. Liu P. Huang Y. Zhou L. Yang Y. Wang H. (2016). Phosphoproteome analysis of invasion and metastasis-related factors in pancreatic cancer cells. 11:e0152280. 10.1371/journal.pone.0152280 27014871 PMC4807880

[B154] Taplin M. E. Manola J. Oh W. K. Kantoff P. W. Bubley G. J. Smith M. (2008). A phase II study of mifepristone (RU-486) in castration-resistant prostate cancer, with a correlative assessment of androgen-related hormones. 101 1084–1089. 10.1111/j.1464-410X.2008.07509.x 18399827

[B155] Thiele I. Clancy C. M. Heinken A. Fleming R. M. T. (2017). Quantitative systems pharmacology and the personalized drug–microbiota–diet axis. 4 43–52. 10.1016/j.coisb.2017.06.001 PMC7493425 32984662

[B156] Tieszen C. R. Goyeneche A. A. Brandhagen B. N. Ortbahn C. T. Telleria C. M. (2011). Antiprogestin mifepristone inhibits the growth of cancer cells of reproductive and non-reproductive origin regardless of progesterone receptor expression. 11:207. 10.1186/1471-2407-11-207 21619605 PMC3125282

[B157] Tran L. N. K. Kichenadasse G. Butler L. M. Centenera M. M. Morel K. L. Ormsby R. J. (2017). The combination of metformin and valproic acid induces synergistic apoptosis in the presence of p53 and androgen signaling in prostate cancer. 16 2689–2700. 10.1158/1535-7163.MCT-17-0074 28802253

[B158] Tsubamoto H. Ueda T. Inoue K. Sakata K. Shibahara H. Sonoda T. (2017). Repurposing itraconazole as an anticancer agent (Review). 14 1240–1246. 10.3892/ol.2017.6325 28789339 PMC5529765

[B159] Turanli B. Gulfidan G. Arga K. Y. (2017). Transcriptomic-guided drug repositioning supported by a new bioinformatics search tool: geneXpharma. 21 584–591. 10.1089/omi.2017.0127 29049014

[B160] Uhlen M. Zhang C. Lee S. Sjöstedt E. Fagerberg L. Bidkhori G. (2017). A pathology atlas of the human cancer transcriptome. 357:eaan2507. 10.1126/science.aan2507 28818916

[B161] Ummanni R. Mundt F. Pospisil H. Venz S. Scharf C. Barett C. (2011). Identification of clinically relevant protein targets in prostate cancer with 2D-DIGE coupled mass spectrometry and systems biology network platform. 6:e16833. 10.1371/journal.pone.0016833 21347291 PMC3037937

[B162] Vanhaelen Q. Mamoshina P. Aliper A. M. Artemov A. Lezhnina K. Ozerov I. (2017). Design of efficient computational workflows for in silico drug repurposing. 22 210–222. 10.1016/j.drudis.2016.09.019 27693712

[B163] Vidal A. C. Howard L. E. Moreira D. M. Castro-Santamaria R. Andriole G. L. Freedland S. J. (2015). Aspirin, NSAIDs, and risk of prostate cancer: results from the REDUCE study. 21 756–762. 10.1158/1078-0432.CCR-14-2235 25520389 PMC4334741

[B164] Wang M. Shim J. S. Li R. J. Dang Y. He Q. Das M. (2014). Identification of an old antibiotic clofoctol as a novel activator of unfolded protein response pathways and an inhibitor of prostate cancer. 171 4478–4489. 10.1111/bph.12800 24903412 PMC4209153

[B165] Wang Y.-C. Chao T.-K. Chang C.-C. Yo Y.-T. Yu M.-H. Lai H.-C. (2013). Drug screening identifies niclosamide as an inhibitor of breast cancer stem-like cells. 8:e74538. 10.1371/journal.pone.0074538 24058587 PMC3776833

[B166] Whitburn J. Edwards C. M. Sooriakumaran P. (2017). Metformin and prostate cancer: a new role for an old drug. 18:46. 10.1007/s11934-017-0693-8 28444639 PMC5405102

[B167] Würth R. Thellung S. Bajetto A. Mazzanti M. Florio T. Barbieri F. (2016). Drug-repositioning opportunities for cancer therapy: novel molecular targets for known compounds. 21 190–199. 10.1016/j.drudis.2015.09.017 26456577

[B168] Xia Q. Sung J. Chowdhury W. Chen C.-L. Höti N. Shabbeer S. (2006). Chronic administration of valproic acid inhibits prostate cancer cell growth in vitro and in vivo. 66 7237–7244. 10.1158/0008-5472.CAN-05-0487 16849572

[B169] Xia W. Lan X. Lv J. Ma J. Chen W. Gao L. (2016). Valproic acid (VPA) suppresses the expression of SMAD4 in prostate carcinoma by up-regulating miR-34a. 9 20466–20473.

[B170] Xu T. Brandmaier S. Messias A. C. Herder C. Draisma H. H. M. Demirkan A. (2015). Effects of metformin on metabolite profiles and LDL cholesterol in patients with type 2 diabetes. 38 1858–1867. 10.2337/dc15-0658 26251408

[B171] Xu X. Shen J. Mall J. W. Myers J. A. Huang W. Blinder L. (1999). In vitro and in vivo antitumor activity of a novel immunomodulatory drug. 58 1405–1413. 10.1016/S0006-2952(99)00228-2 10513984

[B172] Xu Y. Fang F. Sun Y. St Clair D. K. St Clair W. H. (2010). RelB-dependent differential radiosensitization effect of STI571 on prostate cancer cells. 9 803–812. 10.1158/1535-7163.MCT-09-1001 20371728 PMC2852498

[B173] Yizhak K. Chaneton B. Gottlieb E. Ruppin E. (2015). Modeling cancer metabolism on a genome scale. 11 817–817. 10.15252/msb.20145307 26130389 PMC4501850

[B174] Yizhak K. Gabay O. Cohen H. Ruppin E. (2013). Model-based identification of drug targets that revert disrupted metabolism and its application to ageing. 4:2632. 10.1038/ncomms3632 24153335

[B175] Yo Y.-T. Lin Y.-W. Wang Y.-C. Balch C. Huang R.-L. Chan M. W. Y. (2012). Growth inhibition of ovarian tumor-initiating cells by Niclosamide. 11 1703–1712. 10.1158/1535-7163.MCT-12-0002 22576131

[B176] Yoshimura R. Sano H. Masuda C. Kawamura M. Tsubouchi Y. Chargui J. (2000). Expression of cyclooxygenase-2 in prostate carcinoma. 89 589–596. 10.1002/1097-0142(20000801)89:3<589::AID-CNCR14>3.0.CO;2-C 10931458

[B177] Yu S. Yang X. Zhu Y. Xie F. Lu Y. Yu T. (2015). Systems pharmacology of mifepristone (RU486) reveals its 47 hub targets and network: comprehensive analysis and pharmacological focus on FAK-Src-Paxillin complex. 5:7830. 10.1038/srep07830 25597938 PMC4297966

[B178] Zhang C. Hua Q. (2016). Applications of genome-scale metabolic models in biotechnology and systems medicine. 6:413 10.3389/fphys.2015.00413 PMC4703781 26779040

[B179] Zhang H. Qian D. Z. Tan Y. S. Lee K. Gao P. Ren Y. R. (2008). Digoxin and other cardiac glycosides inhibit HIF-1alpha synthesis and block tumor growth. 105 19579–19586. 10.1073/pnas.0809763105 19020076 PMC2604945

[B180] Zhang Q. Wang S. Yang D. Pan K. Li L. Yuan S. (2016). Preclinical pharmacodynamic evaluation of antibiotic nitroxoline for anticancer drug repurposing. 11 3265–3272. 10.3892/ol.2016.4380 27123101 PMC4841112

[B181] Zhang W. Edwards A. Fan W. Flemington E. K. Zhang K. (2012). MiRNA-mRNA correlation-network modules in human prostate cancer and the differences between primary and metastatic tumor subtypes. 7:e40130. 10.1371/journal.pone.0040130 22768240 PMC3387006

[B182] Zhu C. (2013). “Aldose reductase inhibitors as potential therapeutic drugs of diabetic complications,” in , ed. Oguntibeju O. (Rijeka: InTech).

